# The sound of smell: crossmodal correspondences between sound and odor

**DOI:** 10.3389/fpsyg.2026.1890636

**Published:** 2026-09-18

**Authors:** Michèle El Khoury, Olivier Houix, Celine Manetta, Nicolas Misdariis, Patrick Susini

**Affiliations:** 1 International Flavors and Fragrances Inc. (IFF), Neuilly-Sur-Seine, France; 2 STMS UMR 9912 (CNRS/IRCAM/Sorbonne Université), Paris, France

**Keywords:** affective mediation, affects, audition, crossmodal correspondences, emotions, odor, olfaction, sound

## Abstract

**Introduction:**

Crossmodal correspondence plays a subtle yet important role in how we perceive and interpret our environment. Sounds and smells, in particular, might co-occur in our daily lives (e.g., spraying a perfume), yet their interaction remains underexplored. Understanding how these modalities interact can inform multisensory design and sensory marketing. This study investigates whether affects indirectly mediate crossmodal associations between sounds and odors.

**Methods:**

We conducted three experiments. In Experiments 1 and 2, 61 and 60 participants respectively classified 80 sounds and 24 olfactory ingredients according to eight affective categories: *Apathy, Depression, Happiness, Irritation, Relaxation, Sensuality, Stimulation*, and *Stress*. In Experiment 3, 62 participants directly matched 5 groups of sounds to 5 groups of odors, selected based on the results of the first two experiments. This 3rd phase allowed us to compare direct crossmodal associations with the indirect affective mappings from the first two experiments.

**Results:**

Experiments 1 and 2 showed that certain affects, particularly *Stimulation, Stress*, and *Irritation*, were more induced, while others such as *Sensuality* and *Depression* showed weaker or more ambiguous patterns. In the direct matching phase (Experiment 3), some affects (e.g., *Stimulation, Relaxation*) may have predicted crossmodal associations, while others (e.g., *Apathy, Happiness*) were more ambiguous.

**Discussion:**

The results support the hypothesis that affects may function as an indirect link between sound and smell.

## 1 Introduction

The work of Iannis Xenakis “Ç*a sent la musique*” (“*It smells like music*”, 1950) or Yves Saint Laurent's perfume “*Jazz*” (1988) illustrate the creative interaction that exists between the worlds of perfumery and music. In addition, numerous correspondences also exist in the vocabulary used by perfumers: “*notes*,” “*accords*,” “*harmony*” (Piesse, 1867). Nevertheless, despite this shared and metaphorically grounded vocabulary between music and perfumery, there is little information available on crossmodal correspondences between the perception of sounds and odors. Spence (2021) and Spence et al. (2026) note that, despite strong conceptual and linguistic parallels between sound and scent, real-world applications combining these two modalities remain relatively rare. This is partly because odors are difficult to deliver, clear, and synchronize with sound. Where they exist, such applications often rely on storytelling, mood induction, or marketing.

Crossmodal correspondences describe the tendency for a sensory feature to be associated with a feature in a different sensory modality (Deroy et al., 2013; Spence, 2011). Although this phenomenon may seem similar to synesthesia, the two are distinct (Deroy and Spence, 2013; Spence, 2011). Synesthesia is an uncommon condition in which a stimulus elicits an additional perceptual experience not typically present in most people (e.g., seeing a color when hearing a sound; Grossenbacher and Lovelace, 2001; Ward and Mattingley, 2006). These associations are typically automatic and difficult to suppress and are often stable over time (Grossenbacher and Lovelace, 2001; Ward and Mattingley, 2006). In contrast, crossmodal correspondences can be “acquired, malleable, relative, and transitive pairings between sensory dimensions” (Deroy and Spence, 2013, p. 643). Although research on correspondences between audition and olfaction has historically received less attention than other modality pairs, it is a growing area (Deroy et al., 2013; Spence et al., 2024, 2026; Zacharakis, 2025). Existing studies suggest that people often associate high-pitched sounds with sweetness (Crisinel and Spence, 2010; Crisinel and Spence, 2012). In addition, certain sound parameters (e.g., woodwinds, piano, and nature sounds) can enhance freshness-related perceptions in fragrances (Cantu et al., 2022; Rodríguez et al., 2024). Recently, Zacharakis (2024) reported timbre–aroma correspondences between complex synthetic tones and aromatic stimuli in musically trained listeners, and Zacharakis (2025) showed that shared semantic qualities (e.g., brightness and freshness) help explain these crossmodal associations.

Spence (2011) proposed three types of general crossmodal correspondences: *structural correspondences*, which arise from innate neural mechanisms or shared sensory coding (e.g., loudness and visual brightness both linked to the intensity of the stimulus); *statistical correspondences*, which develop through experience with consistent environmental pairings (like large objects producing low-pitched sounds); and *semantic correspondences*, which stem from language development, where certain descriptive terms (e.g., “high” and “low”) are applied across sensory dimensions, creating associations based on linguistic overlap. Similarly, Deroy et al. (2013) focused on crossmodal correspondences between odors and other features such as musical notes or geometrical shapes. They hypothesized that the correspondences that include smell cannot be fully explained by associative learning or metaphorical mappings. Instead, these associations may arise from structural determinants in perception, involving *amodal* dimensions, *indirect* emotional links, or *transitive* mappings across modalities. In fact, the *amodal* hypothesis proposes that odors and sounds may be linked through sensory dimensions common to both modalities, such as perceived intensity or temporal unfolding over time. In audition, temporal evolution may refer to the temporal envelope of a sound (e.g., attack, decay, sustain, and release). In olfaction, it may refer to changes in odor perception over time resulting from differences in odorant volatility, as commonly described by the progression from top to heart and base notes in fragrances (i.e., olfactory pyramid). The *indirect* hypothesis proposes that affective similarities, such as pleasantness, may mediate indirectly these associations. Finally, the *transitivity* hypothesis argues that correspondences may spread through networks: if pitch is associated with angularity, and angularity with certain odors, then pitch may also become associated with those odors.

In the present study, instead of studying music-perfume associations, we focus on sound-odor correspondences, that is, the association between olfactory ingredients and short elementary sounds varying in pitch and timbre. Timbre is a multidimensional auditory attribute that characterizes sound qualities (Acoustical Society of America., n.d.; Peeters et al., 2011). It is usually characterized by acoustic and perceptually based descriptors computed directly from the audio signal, such as spectral centroid (“brightness”), spectral flux, temporal envelope/attack time, etc. (Caclin et al., 2005; McAdams et al., 1995; Peeters et al., 2011). These acoustic descriptors often support instrument identification (Acoustical Society of America., n.d.). Musical sequences involve higher-level structures such as melody, harmony, rhythm, and culturally learned conventions, which can introduce high-level cognitive, semantic and narrative associations. These factors make it more difficult to disentangle perceptual and affective mechanisms underlying sound–odor correspondences. Sound, in our approach, may be considered closer to individual olfactory ingredients at the level of raw perceptual quality. Both are multifaceted sensory attributes that can be examined independently of large scale structural or cultural organization. In fact, single odorants show measurable correlations between perceptual qualities and molecular properties (Keller and Vosshall, 2016). In contrast, fragrances, like musical compositions, usually layer these basic sensory qualities within culturally meaningful structures, drawing on shared associations, aesthetic codes, and symbolic references that go beyond raw perception. Focusing on sounds and odors allowed us to reduce some higher-level narrative and culturally learned associations that are more likely to occur with full musical excerpts and composed fragrances.

Classic crossmodal correspondences between sound and vision such as pitch–brightness and loudness–intensity are often explained by shared structural or amodal dimensions. However, these explanations are difficult to apply to the sound-odor correspondences, as neither can be reduced to a simple, orderly physical dimension, making sensory pairing difficult both methodologically and conceptually. This motivates the exploration of indirect mechanisms, such as affective mediation as proposed by Deroy et al. (2013), which provides a conceptually stronger starting point. Indeed, affects play a central role in both auditory and olfactory experiences (Holley, 1999; Juslin and Laukka, 2004; Korsmit et al., 2023), thus by projecting sound and odor into a shared affective space, we leverage a higher-level dimension that is well-documented, culturally robust, and psychologically meaningful. This approach allows us to examine whether affective similarity contributes to crossmodal associations, alongside possible semantic or structural influences. Following Levitan et al. (2015), we treat sound-odor correspondences as affect-mediated to show that match scores are strongest when odor and sound elicit similar affective profiles.

Affect is the general term covering all forms of evaluative—or valenced (i.e., positive/negative)—states, such as emotions, feelings, or moods (Lochner, 2016; Scherer, 2005). Over time, different theories have emerged to explain the nature of affect. For instance, the discrete theory proposes that a limited set of basic affects (i.e., anger, contempt, disgust, etc.) are innate, universal, from which other affective categories derive (Ekman, 1992; Izard, 1992). In contrast, the dimensional theory argues that affects share a common structure and primarily differ along continuous dimensions, such as valence, and arousal/activation, or valence, tension arousal, and energy arousal (Russell, 1980; Schimmack and Grob, 2000). Modality-specific affective models can capture distinct affects for each sensory modality, which might differ from generalized models that describe everyday affective experiences (Porcherot et al., 2010; Zentner et al., 2008). For example, Zentner et al. (2008) introduced the Geneva Emotional Music Scale (GEMS) with nine music-specific affects like *Nostalgia, Wonder*, and *Power*. Cowen et al. (2020) identified 13 emotional categories across different cultures, including *Beauty* and *Calmness*. In the olfactory domain, the Geneva Emotion and Odor Scale (GEOS; Porcherot et al., 2010) categorizes odor-elicited affects into six different dimensions like *Relaxation, Sensuality*, and *Refreshment*. Churchill and Behan (2010) identified clusters like *Stressful, Stimulated*, and *Relaxed* as a response to olfactory stimuli.

Here, we structured our research using Warrenburg's (2002) *MoodMap*, developed at International Flavors and Fragrances Inc. (IFF) as an olfactory alignment of *Russell's Circumplex Model of Affect* (Russell, 1980). Russell's model organizes affects along two dimensions—valence (pleasant vs. unpleasant) and arousal (high vs. low activation)—forming a circular continuum where adjacent affects are similar, and opposites are distinct. *MoodMap* builds on this topology by mapping olfactory ingredients within the valence–arousal space and grouping them into eight affects (clockwise display): *Stimulation, Happiness, Sensuality, Relaxation, Apathy, Depression, Irritation*, and *Stress*. Although MoodMap has been described in the published literature (Warrenburg, 2002), we used it here as a shared affective framework, that is, a set of affect categories linked to Russell's valence–arousal model, rather than as an independently validated psychometric instrument. This choice was motivated by three reasons: (1) MoodMap maintains theoretical continuity with Russell's model; (2) it regroups affects common to other affect models; and (3) its circular layout provides a reference organization for our study, allowing us to compare the organization of responses to sound and odors against this expected model. Finally, we focus on induced emotions (i.e., what participants feel; the internal affect loci) rather than perceived emotions (i.e., what stimuli express; the external affect loci) to quantify the direct affective impact of sound and odors (Gabrielsson, 2001; Juslin and Laukka, 2004; Korsmit et al., 2023; Song et al., 2016). In fact, a sound or a smell can be sad (external affect loci), but people may still feel happy when listening or smelling it (internal affect loci). In this study, we therefore ask participants to report only their internal affect loci, that is, how the stimuli *make them feel*. Using the MoodMap also involved a trade-off. Modality-specific models, such as GEOS (Porcherot et al., 2010) for odors and GEMS (Zentner et al., 2008) for music, may capture different affects that are less visible in a shared framework. Thus, using *MoodMap* may have reduced sensitivity to some modality-specific affects, while allowing us to test whether sounds and odors could be organized within the same affective space.

Consequently, we hypothesize that affective dimensions may constitute a shared representational space through which sound and odors become associated. By testing whether affective responses mediate sound–odor correspondences, this study aims to clarify the mechanisms underlying crossmodal associations, to extend crossmodal associations to underexplored dimensions related to sound and odor, and to provide a theoretically grounded framework for multisensory design.

To summarize, the present study is designed to test the indirect, affective hypothesis of sound–odor correspondences proposed by Deroy et al. (2013). First, we map the affective profiles elicited by sounds (Experiment 1) and by odors (Experiment 2) into two modality-specific affective spaces. Second, we assess direct sound–odor associations (Experiment 3). Finally, we examine whether these associations can be explained by affective similarity across modalities. If affective mediation accounts for sound–odor correspondences, then sounds and odors that induce similar affective profiles in Experiments 1 and 2 should be more strongly associated in Experiment 3.

## 2 Experiments 1 and 2: sound-affect and odor-affect studies

Hereafter, we will call Experiment 1 the evaluation of the relationship between sound and affects; Experiment 2 the evaluation of the affective associations of odors.

### 2.1 Participants

Participants were required to be French-speaking, to report a normal sense of smell and hearing, and to have no diagnosed psychiatric, neurological, or affective conditions. No specific expertise or preferences in olfaction or sound/music were required. For Experiment 2, additional exclusion criteria included having a cold, being a smoker, being pregnant, or wearing perfume on the day of the experiment. Participants were excluded if their response times were abnormally short, in other words, we excluded the participants that finished in less than 30 min in Experiment 1 or less than 1 h in Experiment 2 (i.e., corresponding to half the estimated time).

No formal *a priori* power analysis was conducted because there was no directly comparable study combining the same olfactory and auditory stimuli, task, and analyses. The target sample size was therefore determined before data collection based on practical considerations. We aimed to recruit approximately 60 participants per experiment, taking into account the exploratory nature of the study and the length of the sensory tasks. This sample size was comparable to, and slightly larger than, that used by Levitan et al. (2015), who tested 40 participants with 15 odors and 15 musical excerpts.

In Experiment 1, 2 participants were excluded from the analysis due to short response times. Consequently, we obtained rating data from 61 participants [43 females; age range: 18–40 years; mean (*M)* = 24.89; Standard deviation (*SD)* = 5.16].

In Experiment 2, we obtained rating data from 60 participants who did not participate in Experiment 1 (44 females; age range: 18–40 years; *M* = 26.1; *SD* = 5.7).

Participants were recruited through the INSEAD Behavioral Lab. All provided informed consent and received compensation for their participation. The protocol was approved by the INSEAD Institution Review Board (IRB Numbers: 2023-67A & 2023-60A).

### 2.2 Stimuli

#### 2.2.1 Experiment 1—sound

We selected 80 sounds (i.e., 10 per affect) based on a pre-test that we ran with five participants (both experts and non-experts) at IRCAM and IFF. The selected sounds originated from two sources: examples in the *Words4Sounds* lexicon of the SpeaK project (Carron et al., 2017; SpeaK Web, n.d.; Susini et al., 2025) and examples from Korsmit et al. (2023) using the Vienna Symphonic Library (2022) database (Korsmit, 2023). The sounds are short, varied in pitch and timbre and included instrumental, synthetic, vocal, and environmental categories, with durations ranging from 0.1 to 15 seconds (See Supplementary Table 1). In the pre-test, 347 sounds were classified into eight affect labels, and the ten top-ranked sounds for each affect category were retained. We also ensured in the selection both clear affective association and acoustic variability. Variability was analyzed using the Timbre Toolbox (Kazazis et al., 2021; Peeters et al., 2011). We analyzed 54 descriptors from the Short-Term Fast-Fourier Transform (STFT; spectral domain), the partials (harmonic domain), the temporal energy envelope (temporal domain) and the estimated F0 (i.e., pitch). For time-varying spectral and harmonic descriptors, we used the median (*M*) and interquartile range (*IQR*) over the duration of the stimuli as summary statistics. A principal component analysis (PCA) was performed on the 54 standardized descriptors extracted from the 80 stimuli to assess variability in timbre and pitch. The first five components accounted for 63.04% of the total variance (*PC1* = 22.54%, *PC2* = 17.09%, *PC3* = 9.77%, *PC4* = 7.19%, *PC5* = 6.44%), evidencing diversity in sound across the set (See Supplementary Material, Figure 1). Because several sounds had identifiable or semi-identifiable sources, participants may have engaged in causal listening, i.e., listening focused on identifying the source or cause of the sound rather than only its acoustic or timbral properties (Lemaitre et al., 2008). This possibility should be considered in the interpretation of the affective responses.

#### 2.2.2 Experiment 2—odors

We selected 24 ingredients (i.e., 3 per affect) in collaboration with a perfumer from IFF (International Flavors and Fragrances Inc.), based on the *ScentEmotions* internal database (Warrenburg, 2002). *ScentEmotions* is a database, in which more than 1,200 scents were associated with affects, textures, and colors. We prioritized diversity across olfactory families (e.g., floral, woody, musky, amber, spicy, etc.), sensory properties, and availability (See Supplementary Table 2).

### 2.3 Apparatus

All sounds (WAV format) were normalized at −20 LUFS using BS.1770-compliant loudness meter (pyloudnorm; Steinmetz and Reiss, 2021) and listened to through headphones (Beyerdynamic DT 770) at 65 dBA. Each sound was labeled with a random letter followed by 2 random numbers (“L70” for example).

The odors were applied to polyethylene discs, placed in clear small glass bottles. Each bottle was labeled with a random letter followed by 2 random numbers (“D92” for example). After each use, the bottles were stored in an empty fridge to minimize the evaporation of the odorants.

Stimulus presentation and data collection were implemented using an HTML- CSS- and JavaScript-based interface run in Microsoft Edge. Sound stimuli were played *via* the HTML5 audio element.

Data analyses were conducted in Python (v3.12) with the Prince library (Halford, n.d.) for correspondence analysis. Silhouette coefficients were computed using scikit-learn (Version 1.6.1; Pedregosa et al., 2011).

### 2.4 Procedure

The main task was a directed sorting task with replacement as each item could be chosen for different labels. For each affect, participants selected stimuli that *induced* (i.e., elicited or made them feel) the corresponding affective state. The experiment was organized according to Warrenburg's *MoodMap* (2002). Eight core affects were tested: *Apathy, Depression* (presented as “*Sadness*”)*, Happiness, Irritation, Relaxation, Sensuality, Stimulation*, and *Stress*. In each trial, participants were presented with one affect label and all stimuli. They selected the stimuli that best induced the target affect. Participants were instructed to select at least 5 out of 80 sounds for Experiment 1 and between 2 and 5 odors of 24 for Experiment 2. Because the same stimulus could be selected for more than one affect across trials, the task produced multi-label binary responses.

We decided to present the word “Sadness” instead of “*Depression*” during the experiment to facilitate definition and avoid triggering language, but this choice should be considered when interpreting the *Depression* category, because “*Sadness*” and “*Depression*” may not be understood as identical affective states. In addition, synonyms for each affect label were provided based on a literature review (Churchill and Behan, 2010; Cowen et al., 2020; Delplanque et al., 2012; Ekman, 1992; Izard, 1992; Juslin and Laukka, 2004; Plutchik, 1980; Porcherot et al., 2010; Russell, 1980; Scherer, 2005; Schimmack and Grob, 2000; Warrenburg, 2002; Zentner et al., 2008).

Prior to the experiments, participants completed two standardized validated questionnaires in French: (1) *PANAS* (Watson et al., 1988; French wording: Nicolas et al., 2014) to assess current mood. Only the standard Positive Affect and Negative Affect scores were calculated and used in the analyses. Along with (2) *PAQ* (Preece et al., 2018; French version: Luminet et al., 2021) to measure alexithymia, that is, difficulty in identifying, describing, and evaluating emotions. Before the main task, participants completed a short familiarization phase. In Experiment 1, all sounds were presented on the same page, and participants had to listen to each sound in full. In Experiment 2, to avoid olfactory saturation, no separate full odor familiarization step was added. Participants were instead asked to take additional time during the first affect block to smell all odors and familiarize themselves with the set. Participants could revisit stimuli during the experiments.

Experiment 1 lasted for 1 h total with no breaks. Experiment 2 was divided into two one-hour sessions. During each session, participants had three mandatory breaks (two of 2 min and one of 5 min). They were also advised to take a break and step outside anytime their nose felt saturated. They were instructed to unscrew the bottles, take small breaths, close the bottle, then place their nose in their elbow, and pause for 2 s (two breaths) between two odors. The interface was designed to ensure that participants attended to all stimuli: in Experiment 1, they had to play/listen to all the sounds before moving to the next affect category. In Experiment 2, a timer was included to discourage rushed responses and encourage exposure to all the odors. Affect categories and stimulus order were randomized for each participant and each session to minimize order effects. Additionally, in Experiment 1, sound labels were randomly generated within each affect so that each step presented different overall codes. This was possible because the presentation of the sounds was digital, unlike the odors which had labels on the bottles and a physical presentation.

Both studies took place in the same spacious, well-ventilated room. Experiment 1 was carried out with 10 participants simultaneously, while Experiment 2 was limited to 5 participants at a time to avoid olfactory mixing in the room.

### 2.5 Results of Experiments 1 and 2

#### 2.5.1 Selection behavior

Figure 1 shows the distribution of the mean number of selections for sounds [panel **(A)**] and odors [panel **(B)**] per affect. Note that the y-axis scales differ between the two figures because the minimum number of requested stimuli was different (at least 5 out of 80 for sounds vs. 2–5 out of 24 for odors). Therefore, the figures should not be compared directly; rather, the distribution within each figure should be interpreted independently. On average, participants selected between 10 and 13 sounds per affect. *Irritation* (*M* = 12.46, *SD* = 7.99) and *Stress* (*M* = 13.03, *SD* = 9.52) showed slightly higher mean selection rates than the rest. In contrast, participants selected, on average, the fewest sounds for *Sensuality* (*M* = 7.33, *SD* = 4.77). Outliers were identified separately for each affective category using the standard 1.5 × IQR rule. Participants were excluded from subsequent analyses only if they were flagged as outliers in more than three affective categories (i.e., at least half of the total). Using this criterion, Participant 34 was identified as an outlier in seven affective categories, and Participant 13 in four affective categories. Their data were therefore excluded from subsequent statistical analyses to reduce potential noise.

**Figure 1:**
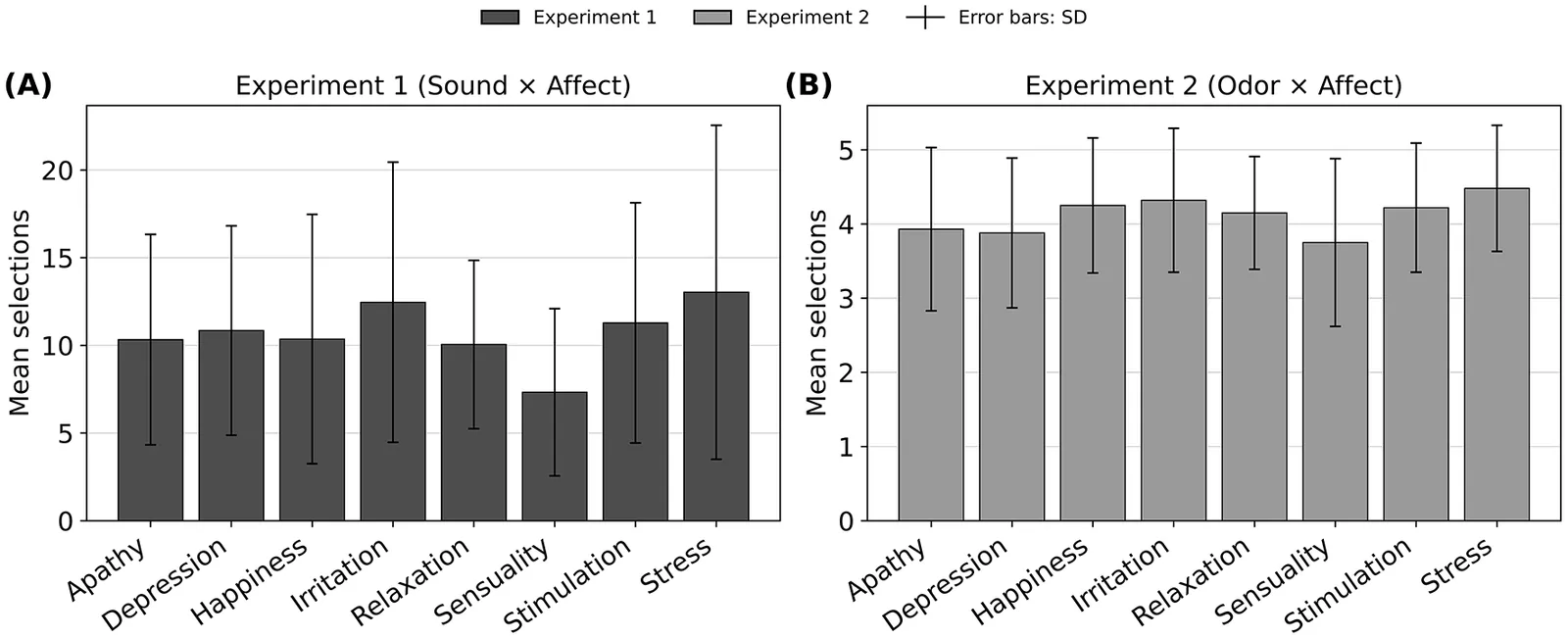
Mean number of selections per affect (± *SD*) in Experiment 1 [sound × affect—Panel **(A)**] and Experiment 2 [odor × affect—Panel **(B)**]. Bars indicate the average number of stimuli selected by participants for each affect; error bars show standard deviations. In Experiment 1, no selection range was imposed, and, in Experiment 2, selections were limited to a 2–5 response range.

Experiment 2 followed Experiment 1. We set an upper limit of five odors to reduce the noise observed in Experiment 1, where some participants selected up to 75% of the sounds per affect. Overall, mean ratings were similar across affects, ranging from 3.75 to 4.48 within the 2–5 selection range. *Sensuality* had the lowest mean selection rate (*M* = 3.75, *SD* = 1.13), while *Stress* had the highest (*M* = 4.48, *SD* = 0.85). Unlike Experiment 1, and due to the imposed limits of two to five odors, no deviation among participants required attention.

The analysis of Krippendorff's alpha coefficient (Krippendorff, 2011), for both experiments, did indicate low agreement across the affects. This outcome is not unexpected, given the subjective nature of affective perception and the sorting design of the task. In fact, Krippendorff's alpha was computed separately for each affective category using binary nominal responses, where selected = 1 and not selected = 0. This approach was used because participants selected stimuli for one affect label at a time, and the same stimulus could be selected for several affects across trials. Alpha was therefore used only as a descriptive measure of inter-participant consistency for each affect. It was not used as a reliability measure for assigning one label to each stimulus. The low alpha values indicate that participants varied in their selections.

#### 2.5.2 Model redundancy

To characterize the affective associations of each stimulus, we constructed a contingency table (See Supplementary Tables 3 and 4) for each experiment that reports the frequency with which each combination stimulus x affect occurs (i.e., how many participants associated each sound or odor with each of the eight affects). In Experiment 1, the number of associations per cell ranges from 0 to 59, corresponding to the total number of participant responses (61 participants minus 2 excluded outliers). Thus, in the contingency table, a value of 0 means that no participant associated a given stimulus with a given affect, whereas a value of 59 means that all participants did so. Experiment 2 follows the same structure, with cell values ranging from 0 to 60.

In Experiment 1, Figure 2 presents the Spearman correlation coefficients (ρ) between the various affective categories induced by the auditory stimuli. These correlations provide insight into potential overlaps or confusions between affects. For instance, a strong positive correlation was observed between *Apathy* and *Depression* (Spearman's ρ = 0.73, *p* < 0.001). Similarly, *Irritation* and *Stress* were highly correlated (Spearman's ρ = 0.80, *p* < 0.001). Conversely, strong negative correlations, such as between *Happiness* and *Depression* (Spearman's ρ = −0.68, *p* < 0.001), highlight clear oppositions in affective responses.

**Figure 2:**
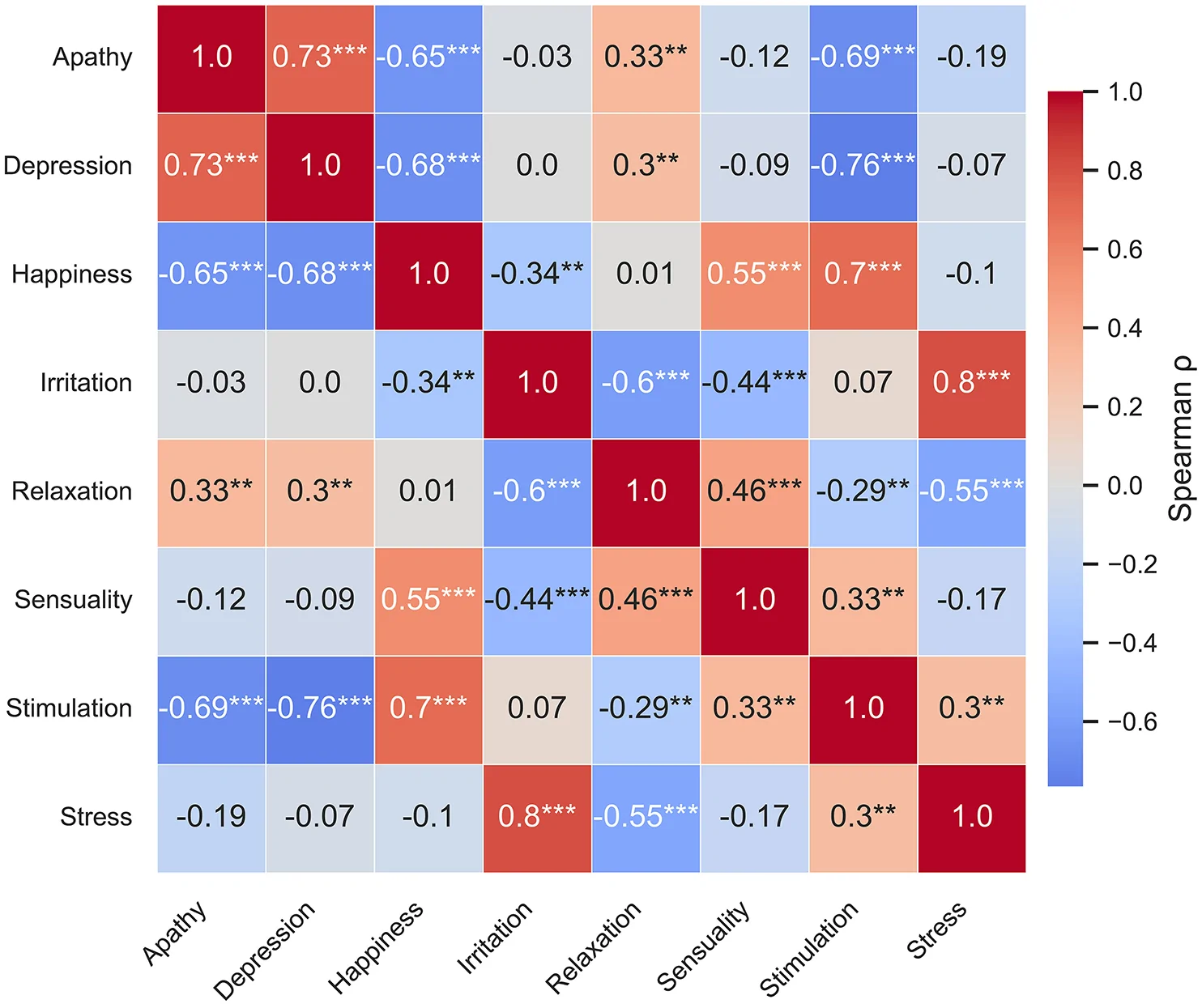
Spearman correlation matrix showing pairwise associations between the eight affect categories evaluated in Experiment 1 (sounds). Each cell represents a Spearman (ρ) coefficient, the colormap reflects the strength and direction of correlations, and asterisks show statistical significance levels. ^*^p < 0.05; ^**^p < 0.01; ^***^p < 0.001.

In Experiment 2, Figure 3 reveals similar patterns. A strong positive correlation was again found between *Apathy* and *Depression* (Spearman's ρ = 0.75, *p* < 0.001), as well as between *Irritation* and *Stress* (Spearman's ρ = 0.90, *p* < 0.001), and between *Relaxation* and *Sensuality* (Spearman's ρ = 0.88, *p* < 0.001), indicating consistent co-induction of these affective states. Strong negative correlations were also observed, particularly between *Irritation* and *Relaxation* (Spearman's ρ = −0.86, *p* < 0.001).

**Figure 3:**
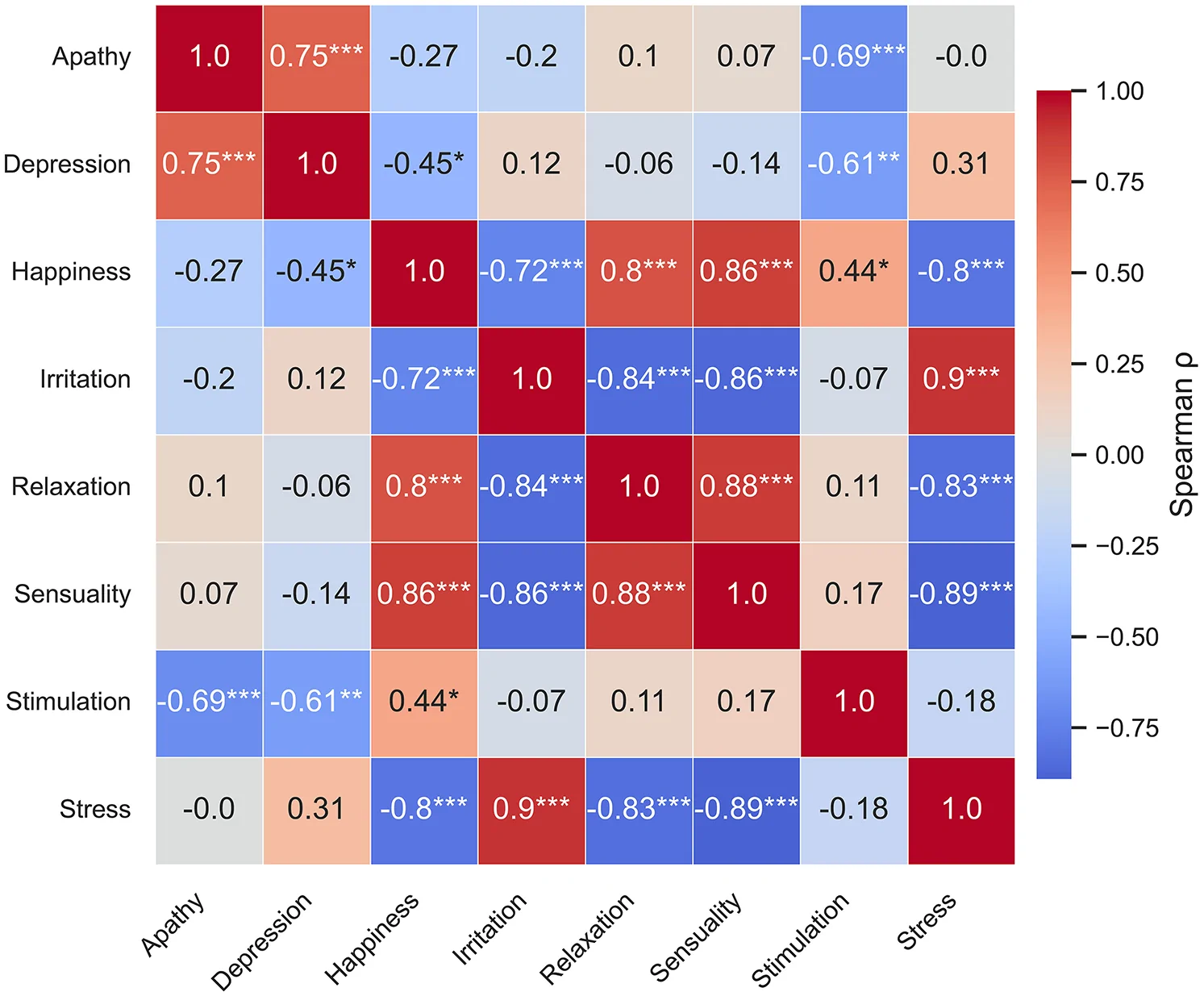
Spearman correlation matrix showing pairwise associations between the eight affect categories evaluated Experiment 2 (odors). Each cell represents a Spearman (ρ) coefficient, the colormap reflects the strength and direction of correlations, and asterisks show statistical significance levels. ^*^p < 0.05; ^**^p < 0.01; ^***^p < 0.001.

To complement the Spearman correlation analysis, we assessed potential redundancy among affective pairs that were significantly and positively correlated. For this purpose, we conducted a chi-square multinomial test to determine whether the distribution of sound–affect associations was statistically indistinguishable between two affects, which would indicate informational redundancy and a lack of unique contribution from each category.

To avoid zero expected frequencies, a value of 0.5 was added to all cells prior to computing the chi-square multinomial test (Haldane–Anscombe correction; Haldane, 1956; Anscombe, 1956). In Experiment 1, only the *Irritation/Stress* pair showed no evidence of a difference between the two affective association profiles, with χ^2^ (79, adjusted *N* = 1471) = 98.77, *p* > 0.05. In Experiment 2, several pairs showed similar results: *Irritation/Stress* [χ^2^(23, *N* = 552) = 16.77, *p* > 0.05], *Depression/Apathy* [χ^2^(23, adjusted *N* = 493) = 19.71, *p* > 0.05], *Sensuality/Happiness* [χ^2^(23, adjusted *N* = 504) = 33.73, *p* > 0.05], and *Sensuality/Relaxation* [χ^2^(23, adjusted *N* = 498) = 24.25, *p* > 0.05].

#### 2.5.3 Mappings of the affects and stimuli

Our analysis will be based on the contingency table (See Supplementary Tables 3 and 4) that also allowed us to derive an emotional profile for each stimulus (the profile is constituted of the association rates with each affect that are presented in the contingency table).

To identify stimuli that might be perceived as emotionally neutral, we conducted chi-square tests for each sound–affect and odor–affect pairing to assess whether the observed distribution of responses significantly deviated from a uniform distribution (equal probability across the eight affect categories). Most sounds and all odors elicited responses that significantly differed from chance, *p* < 0.05. However, three sounds (*Sound 3, 27, and 28*) did not show significant deviation (*ps* = 0.60, 0.20, and 0.20), meaning they have no clear affective association.

To better visualize the relationships between stimuli and affects, we conducted a *Correspondence Analysis (CA*; Beh, 2004; Husson et al., 2011). CA is an exploratory method that uses chi-square distance to project the contingency table into a reduced multidimensional space. To further structure this space, we applied K-means clustering, with the number of clusters determined with the optimal silhouette score (Rousseeuw, 1987). In Experiment 1, the highest average silhouette coefficient was 0.54 and obtained for *k* = *3*, while in Experiment 2, the optimal average silhouette coefficient was 0.61 for *k* = *5*. The complete silhouette profiles are reported in Table 1.

**Table 1:** Average silhouette coefficients for K-means clustering solutions applied to the correspondence analysis maps in Experiments 1 and 2.

k	Experiment 1 (Sound)	Experiment 2 (Odor)
2	0.446	0.499
3	**0.536**	0.522
4	0.482	0.564
5	0.463	**0.609**
6	0.425	0.561
7	0.415	0.531
8	0.430	0.484
9	0.429	0.489

The highest coefficient in each experiment is shown in bold, indicating the selected number of clusters.

In Experiment 1, the variance explained by the first two dimensions of the Correspondence Analysis (CA) is 40.91% for Dimension 1 and 34.28% for Dimension 2. The first cluster included the affects *Irritation* and *Stress;* the second cluster included the affects *Apathy, Depression* and *Relaxation;* and the third cluster included the affects *Sensuality, Happiness*, and *Stimulation*.

All clusters grouped multiple affects, reflecting similarities in their marginal profiles and correlations, which explain their proximity in the CA space. In fact, the inter-affect correlations illustrated in Figure 2 (Section 2.5.2) are also reflected in the 2D affective map. Affects with strong positive correlations, such as *Irritation* and *Stress* (Spearman's ρ = 0.80, *p* < 0.001), or *Apathy* and *Depression* (Spearman's ρ = 0.73, *p* < 0.001), are positioned in close proximity. Conversely, pairs of affects with strong negative correlation, such as *Stimulation* and *Depression* (Spearman's ρ = −0.76, *p* < 0.001), are located diagonally across the map. Also, the neutral sounds mentioned before (i.e., non-significant results in the chi-square calculation; *sounds 3, 27, and 28*) are positioned near the center of the map. The central positioning of neutral sounds reflects their proximity to the average profile, as CA relies on χ^2^ distances weighted by marginal sums. This reinforces their equiprobability and neutrality.

In Experiment 2 (Figure 4), the explained variance was 60.88% for Dimension 1 and 26.12% for Dimension 2. Cluster 1 grouped the affects *Depression* and *Apathy;* cluster 2 grouped *Stimulation* alone*;* cluster 3 grouped the affects *Sensuality, Relaxation*, and *Happiness;* cluster 4 is composed of the affects *Irritation* and *Stress;* finally, cluster 5 is affect-less and is composed solely of stimuli (i.e., the orange cluster in Figure 4). We observe, as well, an almost complete overlap between *Relaxation* and *Sensuality*, consistent with their strong positive correlation (Spearman's ρ = 0.88, *p* < 0.001). Strongly correlated affects, such as *Irritation* and *Stress* (Spearman's ρ = 0.90, *p* < 0.001), or *Apathy* and *Depression* (Spearman's ρ = 0.75, *p* < 0.001), are also located near each other. In contrast, negatively correlated affects, such as *Relaxation* and *Irritation* (Spearman's ρ = −0.84, *p* < 0.001), appear on opposite sides of the map.

**Figure 4:**
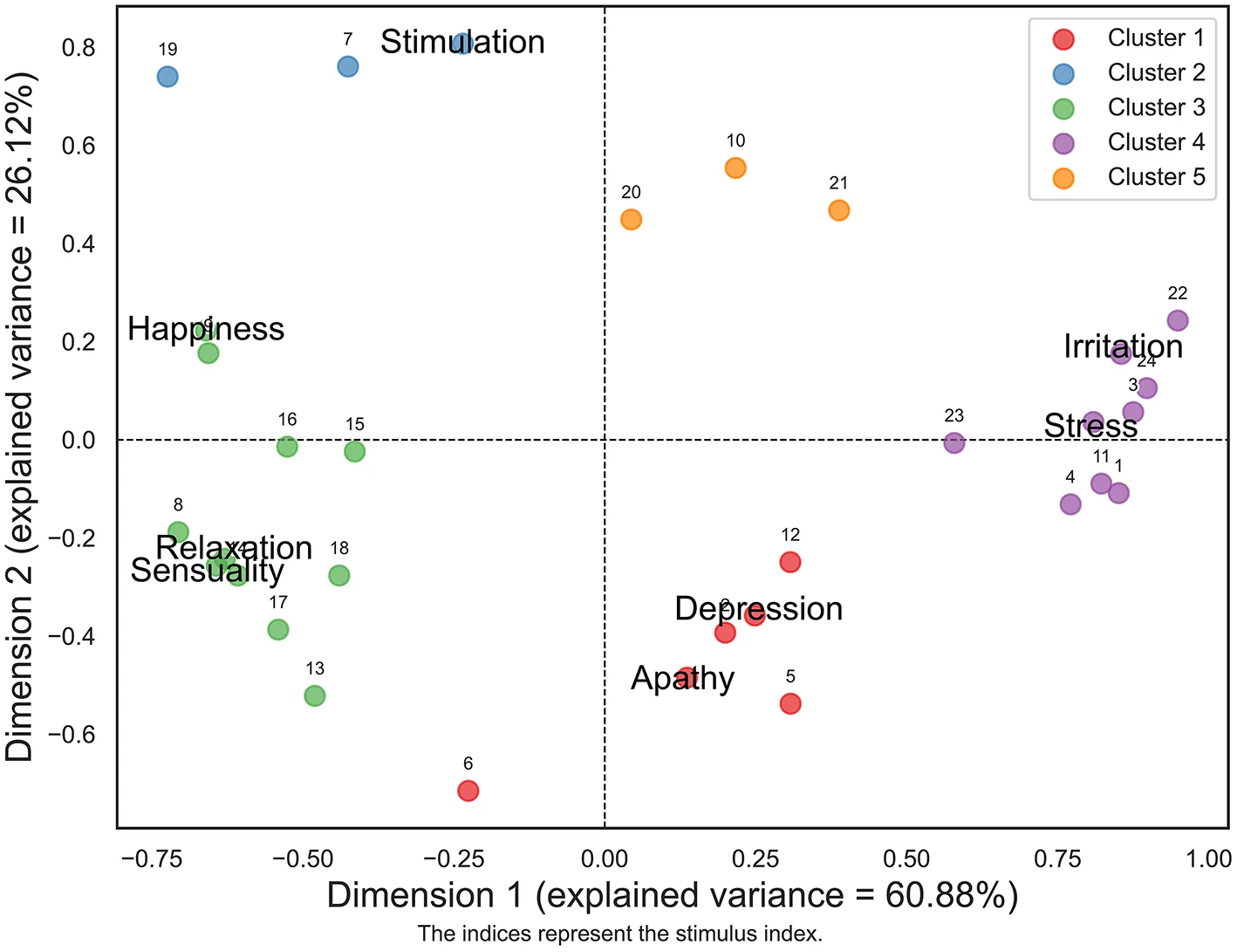
Correspondence analysis (CA) + K-means clustering (5 clusters) of the Experiment 2 Odor × Affect contingency table. The plot shows the CA projection of odors and affect categories onto the first two dimensions (60.88 and 26.12% of explained variance). Five clusters are displayed, based on the optimal K-means solution determined by the average silhouette coefficient (0.61). One cluster contains only stimuli, while the remaining clusters group positively correlated affects (e.g., *Stress–Irritation*).

#### 2.5.4 Individual differences

We computed Spearman's correlation coefficients (ρ) between participants' PANAS Positive Affect and Negative Affect scores, PAQ scores and sub-scores, and participants' number of selections for each affect. The first observation is that none of the correlations across both experiments were particularly strong. Most values were close to zero, indicating weak or negligible relationships between questionnaire scores and selections.

For Experiment 1, the affects showing the highest number of significant correlations (*p* < 0.05) were *Stress* and *Irritation*. These correlations were primarily negative with certain sub scores of alexithymia, showing that individuals with higher alexithymia scores were less likely to select sounds for these affects. Additionally, the positive affect score from the *PANAS* questionnaire showed positive correlations with all affective categories. This implies that participants who reported having a pre-existing good mood were more likely to select more sounds across all affective categories. In reviewing the participants' selection behavior (Section 2.5.1), we noted that some individuals (particularly participants 13 and 34) revealed response patterns that diverged from the rest. However, upon examining their individual questionnaire scores, no notable differences were found compared to the rest of the participants.

A distinct pattern emerged in Experiment 2 regarding the affect *Sensuality*, which showed the highest number of significant correlations (*p* < 0.05). All of these were negative correlations with alexithymia sub scores, that is, individuals with higher alexithymia scores were less likely to associate stimuli with *Sensuality*. However, when examining the results, we observed that most correlations were non-significant, indicating that there is no consistent or strong relationship between questionnaire scores and affective choices for the majority of affect categories.

### 2.6 Discussion of Experiments 1 and 2

Using a directed sorting task, Experiments 1 and 2 investigated how selected sounds and odors map onto eight affective categories based on Warrenburg (2002) *MoodMap*.

#### 2.6.1 Spatial distribution of affects—comparison with Warrenburg's MoodMap

A key contribution of this study lies in its crossmodal comparative approach. We contrasted the spatial organization of affects obtained through correspondence analysis (CA) with the structure proposed in Warrenburg's (2002) *MoodMap*. In Experiment 1 (sounds), the expected circular arrangement of affects was preserved. In Experiment 2 (odors), minor permutations appeared (e.g., *Relaxation* vs. *Sensuality*; *Irritation* vs. *Stress*). Importantly, these shifts remained within their respective theoretical quadrants. Because the affects involved in these permutations are semantically adjacent and conceptually close, their repositioning is not problematic; rather, it indicates that participants perceived them as closely related affective states rather than sharply distinct categories. These subtle relocations therefore do not challenge the validity of the model but instead highlight the need for flexibility when applying affective models across different sensory modalities, populations, and stimuli.

The moderate silhouette values observed in Experiments 1 and 2 suggest that the affective categories are not completely distinct and may partially overlap. However, the selected clustering solutions gave the highest silhouette coefficients among the tested values of *k* and therefore provided the best representation of the sound and odor spaces. The correspondence analysis revealed that some affects clustered with unexpected neighbors, diverging from the model proposed by Warrenburg (2002). For example, in Experiment 1, *Relaxation* clustered with *Apathy* and *Depression*, despite being conceptually distinct in terms of valence (*Relaxation* is a pleasant affect in opposition to *Apathy* and *Depression*). In Experiment 2, *Happiness* is positioned closer to *Relaxation* and *Sensuality*, and further from *Stimulation*. This placement may seem counterintuitive, as *Happiness* is commonly associated with higher arousal, that is, closer to *Stimulation* (Russell, 1980). These deviations reinforce the idea that while these models provide useful foundation, empirical mappings often reveal more complex and context-dependent relationships.

#### 2.6.2 Modality specificity

While some affects, such as *Stimulation* and *Irritation*, were strongly induced in both modalities, others proved more elusive. Notably, *Sensuality* was poorly induced by sounds selected for this study. The top-ranked sounds for *Sensuality* were not exclusive to this affect and showed clear overlap with other categories, with overall lower selection frequencies. This contrasts with Cowen et al. (2020), who found that music can evoke *Desirous* and *Erotic* states. However, as mentioned in the introduction, sound differs fundamentally from music, which involves higher-level structures (melody, complexity, temporal variation, …) referring to high-level cultural/cognitive representations (such as *Desirous/Erotic*). In olfaction, *Depression* was poorly induced, with its associated ingredients showing low and overlapping selection rates, particularly with *Apathy*. These results imply that affect induction can be modality-dependent, and that certain affects may not be equally accessible through all sensory means. It resonates with the work of Porcherot et al. (2010), who proposed nuanced olfactory affect categories such as *Refreshment* and *Sensuality* in the *GEOS* model, and with Zentner et al. (2008), whose *GEMS* model identified music-specific affects like *Wonder and Power*. These models reflect the limitation of using a shared crossmodal affective framework. *Sensuality* is common in olfactory affect models such as *GEOS* (Porcherot et al., 2010*)* but may be less directly accessible through sound. Its weak induction by sounds may therefore reflect modality specificity, and not a limitation of the selected stimuli.

#### 2.6.3 Stimulus-space coverage limitation

In addition, the inability of certain stimuli to induce specific affects may not be solely due to modality but also to stimulus characteristics. Our stimuli may therefore have lacked the complexity needed to induce such affects or failed to activate the representational mechanisms necessary for experiencing them. Factors such as familiarity, identity, and intensity may have influenced the selection process. Because perceived olfactory intensity is difficult to standardize across odorants, we presented each odor at the dilution provided in the ScentEmotions dataset and did not attempt further intensity matching. Consequently, residual differences in perceived intensity may have influenced affective evaluations. This is an important limitation because odor intensity is known to influence affective evaluations (Doty, 1975; Liu et al., 2020). For example, more intense odors may have been more frequently associated with categories such as *Stimulation* or *Irritation*, independently of their qualitative olfactory characteristics. Therefore, part of the affective patterns observed in Experiment 2 may reflect variation in perceived intensity in addition to differences in odor quality. Similarly, loudness influences the structure of the perceptual representation of sound: when loudness is a factor in sound comparison, it generally dominates participants' judgments, particularly in sorting tasks (Susini et al., 2015). Therefore, even though the sounds were normalized at −20 LUFS, it is possible that small differences in loudness affected participants' selection. Furthermore, recall tasks have shown that loudness is an important indicator for recognizing everyday sounds (Susini et al., 2019).

#### 2.6.4 Individual differences

Individual differences in pre-existing mood and alexithymia did not explain affective judgments. While higher alexithymia scores were associated with fewer selections for certain affects (e.g., *Sensuality, Stress*), the overall weak correlations suggest that participants, regardless of emotional awareness or pre-existing mood, evaluated stimuli in a manner close with the broader group. This observation is encouraging, as it supports the robustness of the task and suggests that affective mapping is not heavily influenced by psychological traits. Korsmit et al. (2023) reported moderate associations between pre-existing mood and affective ratings of short instrumental sounds. Additionally, Cecchetto et al. (2023) found that affective ratings of odors were unchanged across alexithymia levels, despite reduced odor awareness and a more indifferent attitude in high-alexithymia individuals.

In summary, identifying these maps that share a similar structuring across sounds and odors allows us to consider testing the indirect hypothesis.

## 3 Sound-odor experiment

Hereafter, we refer to Experiment 3 as the evaluation of direct associations between odors and sounds. Following Experiments 1 and 2, this experiment is part of the evaluation of the indirect hypothesis.

### 3.1 Participants

Participants were required to be French-speaking, to report a normal sense of smell and hearing, and to have no diagnosed psychiatric, neurological, or affective conditions. Additional exclusion criteria included having a cold, being a smoker, being pregnant, or wearing perfume on the day of the experiment. Participants were excluded from the analysis if their response times were abnormally short (i.e., less than 30 min). No specific expertise or preferences in olfaction or sound/music were required.

No participant was excluded. Therefore, a total of 62 different participants (41 females, age range: 18-36 years; *M* = 23.74; *SD* = 3.97) took part in the study.

Participants were recruited through the INSEAD Behavioral Lab. All participants provided informed consent and received compensation. The study protocol was approved by the INSEAD Institutional Review Board (IRB Number: 2023-67A).

### 3.2 Stimuli

The selection of stimuli for Experiment 3 was based on the results of Experiments 1 and 2. The decision was made to represent each affective category with multiple prototypical stimuli rather than a single exemplar. This choice is motivated by Prototype Theory (Rosch, 1973; Rosch and Mervis, 1975), which posits that categories are organized around prototypical examples and exhibit gradual typicality rather than sharp boundaries. Consequently, the sounds and odors most frequently selected in experiments 1 and 2 were retained as typical examples for each affective category and used in Experiment 3. Because these stimuli were selected from the previous experiments, Experiment 3 should be considered a follow-up study rather than a fully independent validation of the affective mapping.

Concretely, stimuli were selected based on the previous experiments' affect maps (Correspondence Analysis and K-means clustering in Figures 4 and 5). We identified the most clearly associated sounds and odors, minimized overlap, and merged affects when necessary. Initially, eight affect groups were planned, each represented by three sounds or two odors; the smaller number of odors was chosen to prevent olfactory overstimulation and saturation. Three odors were excluded because they clustered with no affect (i.e., *ingredients 10, 20* and *21* in the 5^th^ orange cluster in Figure 4). The selection process followed two principles: (1) minimizing overlap by ensuring each stimulus was strongly associated with a single affect, and (2) preserving sensory diversity within each group. Adjustments were made to improve coherence: *Sensuality* (sounds) and *Depression* (odors) were excluded due to low induction rates and overlap with other affects; *Irritation* and *Stress* were merged because of strong correlations and shared stimuli in both modalities (Figures 2 and 3); *Apathy* and *Depression* were also highly correlated (Figures 2 and 3), but *Depression* was excluded due to weak olfactory associations (rather than merged). Ultimately, five affective groups were retained: *Apathy, Happiness, Relaxation, Stimulation*, and *Stress/Irritation*. Each sound group contained three distinct sounds, and each odor group included two different olfactory ingredients, resulting in 25 stimuli (15 sounds and 10 odors; See Supplementary Tables 5 and 6).

**Figure 5:**
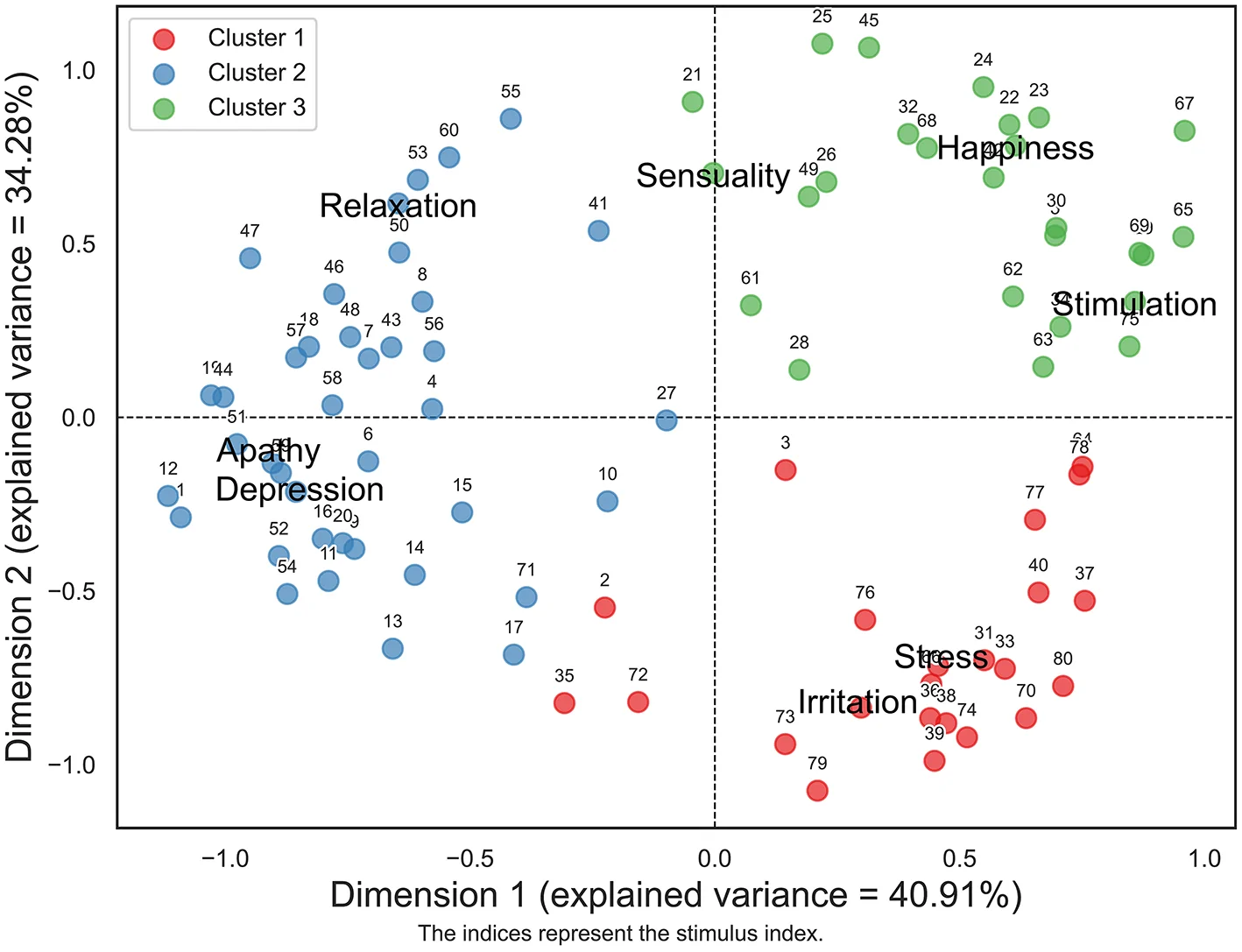
Correspondence analysis (CA) + K-means clustering (3 clusters) of the Experiment 1 contingency table (Sound × Affect). The plot displays the projection of sounds and affect categories onto the first two CA dimensions (40.91% and 34.28% of explained variance). Three clusters are shown, corresponding to the optimal K-means solution determined by the silhouette coefficient (0.54). Cluster positions reflect similarities in marginal profiles and inter-affect correlations (for example, the proximity of *Irritation* and *Stress* or *Apathy* and *Depression*).

### 3.3 Apparatus

The odors were applied again to new polyethylene discs, placed in clear small bottles. The presentation is identical to experiments 1 and 2. Data analyses were conducted in Python (v3.12), cosine similarity and correlation analyses with SciPy (v1.13.1; Virtanen et al., 2020), linear regression models with statsmodels (v0.14.2; Seabold and Perktold, 2010).

### 3.4 Procedure

Each participant was presented with all five odor groups sequentially in a random order. Affects/Emotions/Feelings/etc. were not mentioned at any point, and participants were not given specific criteria for their selections. For each odor group, participants smelled the odors and then listened to all five sound groups, selecting: (1) the sound group that best perceptually matched the smell group (*Top 1*), and (2) the second-best match (*Top 2*). The second choice was included to explore potential secondary associations and was not expected to significantly influence the main results.

Before the matching task, participants completed two familiarization steps. They first listened to all sound groups in full. They then had a 3-min odor familiarization step to smell all odor groups without rushing. During the main matching task, the first presentation of each sound group had to be heard in full; afterward, participants could replay groups freely. Both the groups and the individual stimuli within them were presented in randomized order.

The experiment lasted for a maximum duration of 1 h. Participants had 4 mandatory breaks of 2 min each. They were also advised to take a break and step outside anytime their nose felt saturated. They were instructed to take small breaths, place their nose in their elbow, and pause for 2 s (two breaths) between two odors. The study took place in a spacious, well-ventilated room with only 5 participants at a time to avoid olfactory mix in the room.

### 3.5 Results

#### 3.5.1 Selection behavior

To better understand how participants distributed their selections across sound groups, we calculated the frequency with which individual participants chose each group. The experiment was divided into five trials corresponding to the five groups of odors. In each trial, participants selected two different sound groups for each odor group: one as their first choice (Top 1) and the other as their second choice (Top 2). In total, they made 10 selections, and any single sound group could be chosen up to five times. Looking at the total number of selections per sound group for each participant, we can see that the *Relaxation* sound group emerged as the most frequently chosen category (*M* = 2.63, *SD* = 0.90). In contrast, *Stress/Irritation* received the fewest selections overall (*M* = 1.56, *SD* = 0.95). The remaining groups: *Apathy* (*M* = 2.18, *SD* = 1.11), *Happiness* (*M* = 1.74, *SD* = 0.99), and *Stimulation* (*M* = 1.89, *SD* = 1.03), fell between these two extremes, indicating moderately frequent selection across participants.

In Experiment 3, Krippendorff's alpha was used descriptively to assess the consistency of participants' matching choices. Alpha values per odor group ranged from 0 to 0.2, indicating low inter-participant agreement. This suggests that participants did not always converge on the same sound group choice for each odor group.

#### 3.5.2 Indirect association via affect

##### 3.5.2.1 Confusion matrices

Each participant selected one sound group as their first choice and another as their second choice. These selection rates (= Number of selections / 62 participants) were used to construct two confusion matrices: one for first choices (Top 1) and one for second choices (Top 2; Figure 6).

**Figure 6:**
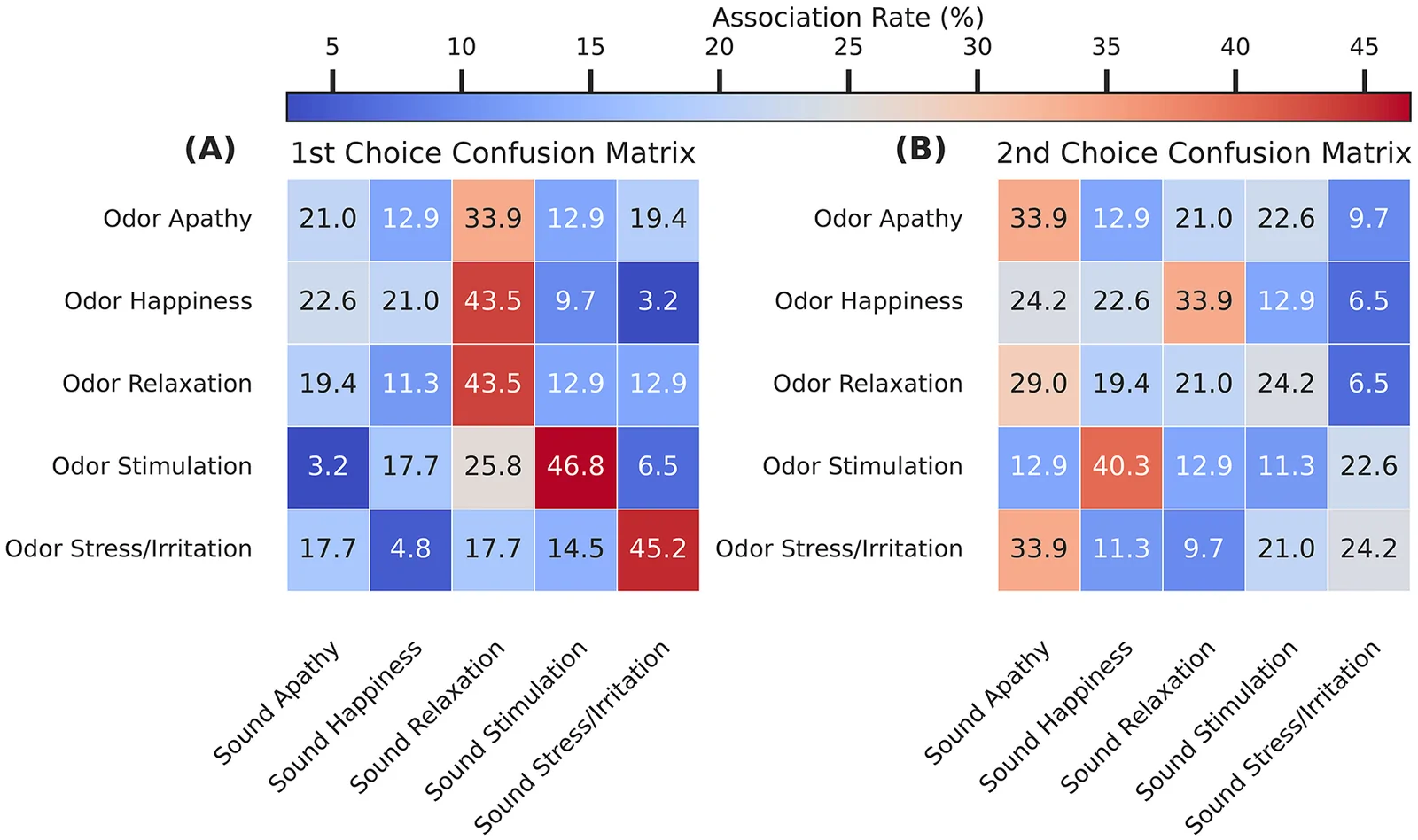
Confusion matrices for participants' first [Panel **(A)**] and second [Panel **(B)**] choices. Each cell shows the percentage of participants who matched an odor group (rows) with a sound group (columns). Warmer colors reflect higher association rates. The first-choice matrix shows strong within-category matches, especially for *Relaxation* and *Stress/Irritation*. The second-choice matrix highlighting secondary crossmodal associations such as *Stimulation*–*Happiness* and *Stress/Irritation*–*Apathy*.

In the first-choice matrix, a prominent diagonal pattern emerged, indicating that participants frequently matched odor and sound groups with congruent affective labels. For instance, the *Relaxation* odor group was most often paired with the *Relaxation* sound group (43.5%), and the *Stress/Irritation* groups were matched at 45.2%. However, we have a mismatch for *Apathy* and *Happiness*, which were most frequently paired with the *Relaxation* sound group (33.9 and 43.5%, respectively).

In the second-choice matrix, the *Apathy* sound group, which was not predominantly selected as a 1^st^ match for the *Apathy* odor group, emerged as its most frequent 2nd pairing. Off-diagonal entries were more pronounced in this matrix. For instance, the *Stimulation* odor group was most associated with the *Happiness* sound group (40.3%), a pairing that is logical due to the nature of these affects. The *Stress/Irritation* odor group was most frequently paired with the *Apathy* sound group (33.9%). The *Relaxation* odor group showed a relatively even distribution across sound groups, with the highest pairing being with *Apathy* (29.0%). Chi-square tests were computed to test whether selections differed from an equiprobability null hypothesis. Under this hypothesis, each of the five sound groups was expected to be selected equally often and each sound group was expected to receive 12.4 selections for each odor group. The *Apathy* odor group showed no significant preference in the 1st matrix, χ^2^ (4, *N* = 62) = 9.12, *p* > 0.05. The *Relaxation* odor group also showed no significant preference in the second-choice matrix χ^2^ (4, *N* = 62) = 8.81, *p* > 0.05.

##### 3.5.2.2 Affective mediation

Since the second-choice selection was an additional selection, it offers interesting insights into secondary affective tendencies. For this reason, it is less reliable for validating our primary hypothesis. Therefore, we focus exclusively on 1st choices to assess the crossmodal mediation of affects.

To examine whether the odor–sound associations observed in Experiment 3 were related to similarities in affective profiles, we compared the affective profiles obtained in Experiments 1 and 2 with the first-choice matching percentages obtained in Experiment 3 (Figure 6).

In Experiments 1 and 2, each stimulus was described by an affective profile based on participants' selections. Here, the selections refer to the percentage of participants who selected a given sound or odor for each affective category. Thus, each sound and each odor had an eight-category affective profile, corresponding to the eight affective categories evaluated in the study. Based on these affective profiles, we selected the best candidates for Experiment 3. We formed five sound groups and five odor groups, each corresponding to one of the five target affects: *Apathy, Happiness, Relaxation, Stimulation*, and *Stress/Irritation*. Each sound group contained three sounds, and each odor group contained two odors. For each sound group and each odor group, we then calculated a mean affective profile. This was done by averaging the number of selections within each group across the eight affective categories. As a result, each sound group and each odor group was represented by one eight-category affective profile. We then compared each odor group with each sound group. Since there were five odor groups and five sound groups, this resulted in 25 odor–sound group pairs (5 x 5). For each pair, we computed the cosine similarity between the odor group's affective profile and the sound group's affective profile. Cosine similarity was used because it measures the similarity in the shape of two vectors. In the present case, higher cosine similarity values indicate that the odor group and sound group had more similar affective profiles. We then compared these cosine similarity values with the first-choice matching percentages from Experiment 3 (Figure 6). For each of the 25 odor group–sound group pairs, the first-choice matching percentage indicated how often participants selected that sound group as the best match for a given odor group. Finally, the relationship between affective cosine similarity and first-choice matching percentage was assessed across the 25 odor group–sound group pairs using Pearson correlation, complemented by a linear regression model with matching percentage as the dependent variable and cosine similarity as the predictor. However, the 25 pairs are not independent observations because each group contributes to five pairs, and matching percentages are constrained to sum to 100% within each odor row. The statistical significance was therefore evaluated with a permutation test. The affect labels were reassigned across odor and sound groups over all 5! × 5! = 14,400 possible relabelings. The cosine-similarity matrix was recomputed at each step, and the correlation was recalculated against the unchanged matching matrix. This procedure preserves both dependency constraints. Because the regression slope and *R*^2^ are monotone functions of *r* under this permutation scheme, the same null distribution provides their significance.

Descriptively, cosine similarity values were high for all congruent odor group–sound group pairs, ranging from 0.90 to 0.96. Among incongruent pairs, the highest cosine similarity values were observed for the *Stimulation* odor group with the *Happiness* sound group (cosine similarity = 0.84); the *Relaxation* odor group with the *Happiness* sound group (cosine similarity = 0.83); and the *Apathy* odor group with the *Relaxation* sound group (cosine similarity = 0.82). Across the 25 odor group–sound group pairs, affective similarity was positively associated with first-choice matching percentage (Pearson's *r* = 0.69). The linear regression model accounted for 48% of the variance in matching percentage (β = 34.75, *p* < 0.001, with *R*^2^ = 0.48). The observed correlation and the results of the linear regression model exceeded the permutation null, with *p* < 0.001 (two-sided, 14,400 permutations; Figure 7).

**Figure 7:**
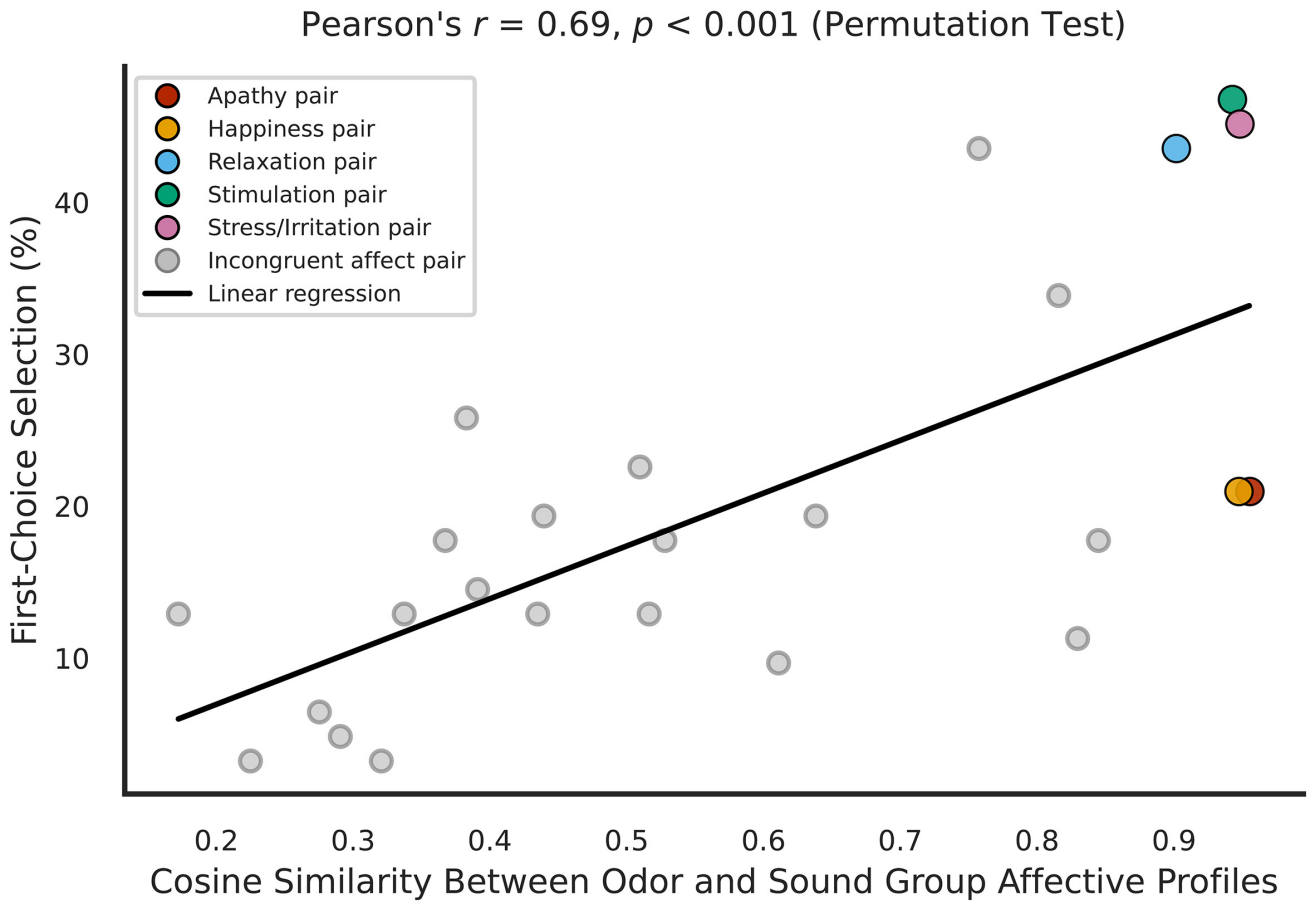
Relationship between affective cosine similarity and first-choice matching percentage for all odor–sound group pairs. Each point represents one of the 25 odor group–sound group pairs. Colored points indicate congruent pairs, in which the odor group and the sound group share the same affective label (e.g., *Relaxation* odor group and sound group); gray points indicate incongruent pairs (e.g., the *Relaxation* sound group and the *Stimulation* odor group). The solid black line represents the linear regression fit across all pairs. A permutation test indicated that higher affective cosine similarity was associated with higher matching percentages (Pearson's *r* = 0.69, *p* < 0.001; two-sided, 14,400 permutations).

### 3.6 Discussion of Experiment 3

To evaluate the hypothesis that crossmodal correspondences can be explained by the indirect hypothesis, Experiment 3 tested whether participants would associate odors and sound stimuli based on affective congruence, without being explicitly guided by affect labels. Overall, the results suggest that affective similarity may play an important role in odor–sound associations.

#### 3.6.1 Evidence of affective mediation in crossmodal correspondences

The present results suggest that odor–sound correspondences in Experiment 3 were partly driven by affective similarity. This pattern suggests that participants' choices may be guided by shared emotional associations. Odor–sound pairs with more similar affective profiles tended to be matched more frequently by participants. This result is consistent with the indirect affective mediation hypothesis of crossmodal correspondences (Deroy et al., 2013). In the present study, this means that an odor and a sound may have been associated because they induced similar affective responses.

The matching matrix also points in this direction. *Relaxation, Stimulation*, and *Stress/Irritation* showed relatively clear odor–sound correspondences. For these categories, odor groups were most frequently matched with sound groups carrying the same affective label. This suggests that these affective categories may have been more clearly represented across both modalities, making them easier to associate. The complementary regression analysis supports this interpretation, indicating that affective similarity accounted for about 48% of the variance in matching percentages. This suggests that affective similarity was a relevant predictor of participants' choices. However, it's important to note that the correlation and regression results should be considered exploratory rather than confirmatory of the indirect hypothesis by Deroy et al. (2013).

#### 3.6.2 Limitations of affective mediation: *Happiness* and *Apathy*

Although affective similarity was related to matching behavior, it did not fully account for participants' choices. A substantial part of the variance remained unexplained. This suggests that other dimensions (e.g., intensity, liking, familiarity, etc.) may also have contributed to odor–sound correspondences. Some of these dimensions may overlap with affect, but they were not fully captured by the eight affective dimensions used in the present analysis. Consequently, the present results do not allow us to exclude alternative mechanisms proposed by Deroy et al. (2013). In particular, the study was not designed to directly evaluate amodal dimensions, nor did it measure links that could support transitive correspondences. Therefore, affective mediation should be interpreted as one plausible contributor to odor-sound correspondences but not as their exclusive explanation.

The limits of affective mediation were also visible in the matching patterns. While *Relaxation, Stimulation*, and *Stress/Irritation* showed relatively clear correspondences, *Happiness* and *Apathy* showed more ambiguous patterns. These categories were less consistently matched with their affectively congruent sound groups. Instead, they appeared closer to neighboring categories, especially *Relaxation*. While most stimuli in Experiment 3 showed well-defined affective profiles, the *Apathy* and *Happiness* groups appear to reflect limits in discriminability rather than a contradiction of the affective mediation hypothesis.

For *Apathy*, the selected stimuli were among the most representative candidates available, but this affect remained close to neighboring categories. In Experiment 1, the CA and correlation matrix (Figures 2 and 5) positioned *Apathy* near *Relaxation* and *Depression*, suggesting limited affective separation. Therefore, in Experiment 3, the *Apathy* odor group did not show a clear first-choice preference for the *Apathy* sound group. This suggests that *Apathy* did not provide a sufficiently distinct affective profile to function as a reliable crossmodal mediator. For *Happiness*, the selected odors were also the most prototypical candidates available from Experiment 2, but they remained close to *Relaxation/Sensuality* in the olfactory affective space (Figure 4). Because participants completed Experiment 3 without affect labels, their choices likely reflected the affective impression actually induced by the odor group rather than its intended label. The predominance of *Relaxation* choices for the *Happiness* odor group therefore suggests that this group was experienced as closer to *Relaxation* than to a distinct *Happiness* profile. Thus, the absence of a *Happiness*–*Happiness* match in Experiment 3 should not be interpreted as evidence that the *Happiness* sound group failed in isolation. Rather, it indicates that the *Happiness* odor group did not provide a sufficiently distinct olfactory equivalent for this affect, and that successful affective mediation requires sufficiently distinct affective profiles in both modalities.

These findings suggest that *Apathy* and *Happiness* represent boundary cases for affective mediation in the present stimulus set. Furthermore, these affects often reflect broader, everyday experiences that are difficult to capture within the constraints of our experimental setting. *Happiness*, for instance, can involve life satisfaction and wellbeing rather than only a momentary sensory pleasure (Diener, 2009). *Apathy* may also have a less distinctive sensory signature, as it is commonly defined as a lack of motivation expressed through reduced goal-directed behavior (Le Heron et al., 2019). Consequently, our deliberate choice to work with sound–odor correspondences, precisely to avoid higher level cognitive, cultural, or narrative representations such as those found in music–fragrance associations, also comes with a limitation. What we gain in control and precise characterization of sound/odor, we lose in our ability to induce more complex and context-dependent affects such as *Happiness* and *Apathy*. This ambiguity does not necessarily contradict the affective mediation account. It suggests that affective mediation may be more effective when affective profiles are clearly differentiated. When the profiles are less distinct, crossmodal matching may become less stable. In this case, participants may rely on other dimensions in addition to affect.

Experiment 3 was built from the affective profiles obtained in Experiments 1 and 2 and relied on prototype-based selections. Our initial aim was to represent all eight affects but Experiments 1 and 2 showed that some categories were strongly overlapping and/or weakly induced. We therefore retained the clearest affective groups to test whether prototypical profiles could support odor–sound matching. This means that Experiment 3 does not fully capture the mixed nature of affective responses. Future work should use larger stimulus sets or continuous ratings to model ambiguous stimuli directly. The merging (e.g., *Stress/Irritation)* and exclusion (e.g., *Depression, Sensuality)* decisions were therefore data-driven and aimed at retaining the clearest affective prototypes. For this reason, Experiment 3 should be interpreted as a follow-up test rather than as a fully independent validation of the affective mapping. Because the retained categories were also the most clearly discriminated in Experiments 1 and 2, Experiment 3 tested matching under favorable conditions. However, participants were new, were not informed about the affective categories, and were not asked to match stimuli based on affect. Thus, the results provide support that affective similarity may play a role in odor–sound correspondences, but they should not be taken as an independent confirmation of the full affective structure.

#### 3.6.3 Group-based vs. item-based protocols

A key change in this protocol was the presentation of stimuli in grouped formats, treating them as units rather than isolated items, consistent with the prototype theory (Rosch, 1973; Rosch and Mervis, 1975). This design choice also contrasts with prior studies on olfactory–auditory correspondences (Levitan et al., 2015; Zacharakis, 2025). This approach was conceptual and practical: crossmodal correspondences between sound and scent are more difficult to establish because these modalities co-occur less frequently in natural environments compared to others (Deroy et al., 2013; Spence, 2011). Unlike vision and audition, these associations are often metaphorical, structural or affectively mediated rather than learned through repeated direct exposure. By presenting multiple items within each affective category, we sought to evoke a richer and more immersive impression, one that participants could intuitively perceive. Of course, grouping comes with interpretive challenges. When stimuli are presented together, it is harder to know whether associations reflect the overall impression or a particularly dominant item. A salient sound or scent could shape the perception of the entire group, skewing responses. Future studies employing item-based designs could help mitigate such comparison biases and reveal the indirect role of affect in shaping crossmodal associations.

## 4 Global discussion

### 4.1 Key findings

In this study, we provide preliminary evidence suggesting that crossmodal associations between odors and sounds may be mediated by affective responses, in line with the indirect hypothesis proposed by Deroy et al. (2013). Specifically, we asked: if a sound and an odor evoke the same affect, are they more likely to be associated, even without explicitly mentioning affect? To evaluate this, we designed three experiments. The first two mapped the affective profiles of sounds and odors respectively, using Warrenburg's (2002) *MoodMap* as a framework. The third experiment evaluated whether participants would associate sounds and odors based on these shared affective profiles, without being told anything about affects. Across three experiments, our findings suggest that affective similarity may help explain sound–odor associations, providing support for the idea that sound–odor correspondences can arise indirectly through shared affective responses. In other words, affect provides a natural shared dimension for two systems with limited common structure, without relying mainly on semantic mediation.

More specifically, across the first two experiments, we observed that most affects, such as *Stimulation, Relaxation*, and *Irritation*, were strongly induced across both modalities. *Sensuality* and *Depression* showed lower associations in the auditory and olfactory domains respectively. These were excluded from the third phase to improve clarity. These findings indicate that while many affective states are intelligible across modalities, some remain modality dependent. Furthermore, observed correlations between certain affects (e.g., *Irritation–Stress, Apathy–Depression*) suggest redundancy within affective categories in both modalities, consistent with Warrenburg (2002) where *Depression* and *Apathy* were grouped as one. Experiment 3 showed that participants might have matched odors and sounds based on shared affective profiles (i.e., *Relaxation, Stimulation*, and *Stress/Irritation)*, even without instructions, supporting potential affective mediation. In contrast, *Happiness* and *Apathy* produced weaker matches, reflecting the limited discriminability of these affects already observed within each modality (Odor for *Happiness* and sound for *Apathy*). Their ambiguity might stem from the difficulty of evoking complex or context-dependent states with sound and odor ingredients. To improve affective discriminability, future studies could use stimuli that minimize automatic semantic or memory-based associations. In the auditory domain, this may involve using abstract or *concrete* sounds inspired by Musique Concrète (Schaeffer, 1952), which dissociate perception from identifiable sound sources and reduce causal listening biases (Lemaitre et al., 2008). In olfaction, selecting less familiar odorants can limit the influence of culturally learned associations and personal memories. Overall, the results suggest that affective similarity might contribute to most odor–sound associations. Importantly, the present study was designed to evaluate the indirect affective hypothesis but doesn't dissociate between indirect, amodal, and transitive mechanisms. While the observed relationship between affective similarity and crossmodal matching supports a role for affective mediation, alternative mechanisms cannot be excluded. For example, amodal dimensions such as perceived intensity or temporal evolution were not directly measured or controlled. Future studies should manipulate these dimensions independently to determine their relative contribution. Another limitation is that the sorting task produced binary selections rather than graded ratings. Nevertheless, the goal of Experiment 1 and 2 was not to measure the intensity of each affective association, but to identify stimuli that could serve as affective prototypes for each category. Non-selection should still be interpreted cautiously. It may reflect lower salience or lower prototypicality, and not the absence of an affective response. This is especially important because the task did not ask participants to rate each stimulus on a continuous affective scale. Future work could complement this prototype-based approach with continuous affect ratings and pairwise continuous ratings between stimuli (Levitan et al., 2015; Palmer et al., 2013), to obtain more graded affective and matching profiles. In addition, it should be noted that inter-rater agreement was low across experiments, but this is commonly observed in affective and multi-label annotation contexts, where judgments are inherently subjective and multiple interpretations may be valid (Du and Hoste, 2025; Marchal et al., 2022). For instance, Korsmit et al. (2023) showed that responses to sound diverge across perceived vs. induced affect and are better captured by dimensional than discrete models. Therefore, the affective profiles should not be interpreted as fixed consensus labels. They should instead be understood as tendencies in how participants associated each stimulus with the eight affective categories. Thus, despite low agreement, the dataset remains valuable for modeling the diversity of affective interpretations.

Importantly, our results reproduced the circular structure of Russell (1980) model of affect, which inspired Warrenburg (2002). For both modalities, the arrangement of affects was organized according to valence and arousal in a circular space where proximity reflects emotional similarity. This is consistent with studies showing that odors (Toet et al., 2020) and sounds (Eerola et al., 2012; Korsmit et al., 2023; McAdams et al., 2017) can be structured within valence–arousal spaces. The reproduction of this configuration across the experiments reinforces affect as a shared dimension, consistent with Deroy et al. (2013) indirect hypothesis.

Prior work has shown that emotional similarity can predict odor–music associations (Levitan et al., 2015; Spence, 2020; Spence et al., 2024, 2026), and that olfactory and auditory stimuli are linked to other modalities through hedonic and emotional dimensions (Di Stefano et al., 2024; Spence, 2011; Ward et al., 2021). These correspondences often rely on diffuse emotional mediation, making them more context-dependent than structural mappings (Liu and Lupyan, 2026; Spence, 2011; Spence et al., 2024). Musical sequences carry melodic, harmonic, rhythmic, and stylistic structures, and fragrances embed rich semantic and subjective content. These higher level factors might obscure low level crossmodal processes. Sound and odor ingredients, by contrast, involve lower-level sensory attributes that may reduce, although not eliminate, the influence of language, identity, and higher-level narrative associations (Keller and Vosshall, 2016; McAdams et al., 1995), making them well suited for affective correspondence routes. Supporting this view, the physicochemical features of odorants have been shown to predict crossmodal associations (i.e., with pitch, shape) even in the absence of semantic cues (Ward et al., 2022). Thus, we extended this framework to sound and odor ingredients—stimuli with fewer explicit semantic cues than full musical excerpts or composed fragrances. We do not assume that semantic or conceptual processes are absent, especially because affective categories are verbally labeled in the experiments. Rather, the use of isolated sounds and single odorants was intended to reduce higher-level narrative and culturally learned associations, not to fully eliminate semantic processing. Overall, our results provide an evaluation of this mechanism, supporting the idea that affect might be a potential mediating factor underlying crossmodal correspondences even between modalities with limited natural co-occurrence.

### 4.2 Conclusions

In summary, the study provides evidence that associations between sound and odor can be driven indirectly by shared affective responses. Across three experiments, we first mapped the affective profiles of sounds and odors, then showed that participants tended to associate sounds and odors with similar affective profiles, even when affect was never explicitly mentioned. By investigating crossmodal correspondences between sound and odor—two modalities that have been relatively less studied together—this work supports the indirect hypothesis proposed by Deroy et al. (2013). The results further suggest that affect induction depends on both sensory modality and stimulus design. In fact, while some affects are reliably elicited, others appear more sensitive to contextual or stimulus-specific factors. Future research could explore additional mediators of crossmodal associations, such as semantic factors or amodal perceptual attributes (e.g., intensity, temporal structure). It would also be important to consider the role of participant diversity, as emotional perception and subjective responses are influenced by age, gender, and cultural background (Cowen et al., 2020; Wan et al., 2014). Finally, these findings have implications for the design of interactive multisensory systems, where leveraging affective correspondences across modalities may support more coherent and engaging multisensory experiences and enhance the overall quality.

## Data Availability

The original contributions presented in the study are included in the article/Supplementary Material. The datasets underlying the full olfactory stimulus sets - including the identity of the individual chemical identities, ingredient names, supplier information, concentrations, dilution levels and item-level stimulus files of the 24 olfactory ingredients drawn from IFF's ScentEmotions database - are not readily available. The olfactory ingredients and the associated item-level data are proprietary to IFF (International Flavors & Fragrances). All of the above are subject to confidentiality and intellectual-property restrictions. Requests to access these datasets, or further inquiries, should be directed to the corresponding author.
