# Supplementary material for: The sound of smell: crossmodal correspondences between sound and odor
Source: Front Psychol. 2026 Sep 18;17:1890636. doi: 10.3389/fpsyg.2026.1890636 (PMC13631861; doi:10.3389/fpsyg.2026.1890636)
Supplement: Supplementary file 1 [file Supplementary_File_1.docx]

**Supplementary Figure 1.** Principal Component Analysis (PCA) loadings of the 54 acoustic descriptors extracted from the 80 sound stimuli using the Timbre Toolbox (Peeters et al., 2011; Kazazis et al., 2021). Descriptors were computed from the spectral (Short-Term Fast-Fourier Transform), harmonic (partials), and temporal (energy envelope) domains, as well as from the estimated fundamental frequency (F_0_). For time-varying spectral and harmonic descriptors, the median (*Median*) and interquartile range (*IQR*) across the duration of each stimulus were used as summary statistics. Each point represents one descriptor projected onto the first two principal components (*PC1* = 22.54%, *PC2* = 17.09%), which together account for 39.63% of the total variance across the stimulus set. The spread of descriptors across both dimensions illustrates the acoustic diversity of the 80 sound stimuli.
