# Supplementary material for: The sound of smell: crossmodal correspondences between sound and odor
Source: Front Psychol. 2026 Sep 18;17:1890636. doi: 10.3389/fpsyg.2026.1890636 (PMC13631861; doi:10.3389/fpsyg.2026.1890636)
Supplement: Supplementary file 2 [file Table_1.docx]

**Supplementary Table 1.** The table provides information about the 80 sound stimuli. Sounds came from examples in the *Words4Sounds* lexicon of the SpeaK project (SpeaKWeb; Susini et al., 2025; Carron et al., 2017) and examples from Korsmit et al. (2023) using the Vienna Symphonic Library (VSL; 2022) database (Korsmit, 2023). The sounds originating from VSL were described according to their instrument family, instrument name, and pitch register. The sounds originating from SpeaKWeb were described according to their targeted timbre descriptor and sound source. Non-applicable or unavailable information was marked as “-”.

| Sound | Database | Sound/Family Source | Duration  (in seconds) | Instrument family | Instrument | Register | Timbre characteristic |
| --- | --- | --- | --- | --- | --- | --- | --- |
| S1 | VSL | Instrumental | 3 | Woodwind | Contrabassoon | 2 | - |
| S2 | VSL | Instrumental | 3 | Woodwind | Oboe | 4 | - |
| S3 | VSL | Instrumental | 3 | Percussion | Timpani | 2 | - |
| S4 | SpeaKWeb | Instrumental | 5 | - | - | - | Artificial |
| S5 | SpeaKWeb | Voice (Female) | 0.27 | - | - | - | Sharp attack |
| S6 | SpeaKWeb | Instrumental | 5 | - | - | - | Constant |
| S7 | SpeaKWeb | Environmental | 15 | - | - | - | Smooth |
| S8 | SpeaKWeb | Synthetic | 5 | - | - | - | Non-rich |
| S9 | SpeaKWeb | Synthetic | 0.12 | - | - | - | Non-round |
| S10 | SpeaKWeb | Synthetic | 5 | - | - | - | Round |
| S11 | VSL | Instrumental | 3 | Brass | Contrabass Tuba | 2 | - |
| S12 | VSL | Instrumental | 3.41 | Brass | Tuba | 3 | - |
| S13 | VSL | Instrumental | 3 | Woodwind | Contrabassoon | 1 | - |
| S14 | VSL | Instrumental | 3 | Strings | Harp | 1 | - |
| S15 | SpeaKWeb | Synthetic | 5 | - | - | - | Progressive attack |
| S16 | SpeaKWeb | Instrumental | 5 | - | - | - | Low |
| S17 | SpeaKWeb | Instrumental | 5 | - | - | - | Smooth |
| S18 | SpeaKWeb | Instrumental | 5 | - | - | - | Dull |
| S19 | SpeaKWeb | Synthetic | 5 | - | - | - | Natural |
| S20 | SpeaKWeb | Instrumental | 5 | - | - | - | Non-nasal |
| S21 | VSL | Instrumental | 3 | Strings | Harp | 5 | - |
| S22 | VSL | Instrumental | 3 | Percussion | Glockenspiel | 7 | - |
| S23 | VSL | Instrumental | 1.60 | Percussion | Xylophone | 5 | - |
| S24 | VSL | Instrumental | 3 | Percussion | Vibraphone | 6 | - |
| S25 | SpeaKWeb | Instrumental | 8.01 | - | - | - | Ascending |
| S26 | SpeaKWeb | Voice | 2.69 | - | - | - | Progressive attack |
| S27 | SpeaKWeb | Synthetic | 5 | - | - | - | Fluctuating |
| S28 | SpeaKWeb | Voice (Female) | 5 | - | - | - | Rich |
| S29 | SpeaKWeb | Synthetic | 5 | - | - | - | Rich |
| S30 | SpeaKWeb | Voice (Female) | 0.52 | - | - | - | Round |
| S31 | VSL | Instrumental | 3 | Brass | Piccolo Trumpet | 6 | - |
| S32 | VSL | Instrumental | 3 | Percussion | Celesta | 7 | - |
| S33 | SpeaKWeb | Voice (Female) | 2.94 | - | - | - | Bright |
| S34 | SpeaKWeb | Instrumental | 9.31 | - | - | - | Fluctuating |
| S35 | SpeaKWeb | Environmental | 5.63 | - | - | - | Nasal |
| S36 | SpeaKWeb | Voice (Female) | 3.68 | - | - | - | Nasal |
| S37 | SpeaKWeb | Synthetic | 5 | - | - | - | Rough |
| S38 | SpeaKWeb | Voice (Male) | 5.30 | - | - | - | Strident |
| S39 | SpeaKWeb | Instrumental | 5.41 | - | - | - | Strident |
| S40 | SpeaKWeb | Environmental | 3.14 | - | - | - | Tonal |
| S41 | VSL | Instrumental | 3 | Percussion | Gong | 5 | - |
| S42 | VSL | Instrumental | 3 | Percussion | Crotales | 7 | - |
| S43 | SpeaKWeb | Environmental | 2 | - | - | - | Progressive attack |
| S44 | SpeaKWeb | Instrumental | 5 | - | - | - | Warm |
| S45 | SpeaKWeb | Instrumental | 8.08 | - | - | - | Descending |
| S46 | SpeaKWeb | Synthetic | 5 | - | - | - | Low |
| S47 | SpeaKWeb | Instrumental | 5 | - | - | - | Non-warm |
| S48 | SpeaKWeb | Synthetic | 4 | - | - | - | Resonant |
| S49 | SpeaKWeb | Instrumental | 8.04 | - | - | - | Rich |
| S50 | SpeaKWeb | Instrumental | 4.93 | - | - | - | Round |
| S51 | VSL | Instrumental | 3 | Woodwind | Bassoon | 4 | - |
| S52 | VSL | Instrumental | 3 | Woodwind | Bass Clarinet | 2 | - |
| S53 | VSL | Instrumental | 3 | Strings | Harp | 3 | - |
| S54 | VSL | Instrumental | 2.6 | Strings | Cello | 3 | - |
| S55 | VSL | Instrumental | 3 | Percussion | Celesta | 3 | - |
| S56 | SpeaKWeb | Instrumental | 5 | - | - | - | Noisy |
| S57 | SpeaKWeb | Voice (Female) | 4.90 | - | - | - | Warm |
| S58 | SpeaKWeb | Voice (Male) | 4.43 | - | - | - | Warm |
| S59 | SpeaKWeb | Instrumental | 5 | - | - | - | Warm |
| S60 | SpeaKWeb | Instrumental | 7.74 | - | - | - | Non-rich |
| S61 | VSL | Instrumental | 3 | Brass | Piccolo Trumpet | 5 | - |
| S62 | VSL | Instrumental | 2.40 | Strings | Harp | 7 | - |
| S63 | SpeaKWeb | Voice (Female) | 6.23 | - | - | - | Ascending |
| S64 | SpeaKWeb | Synthetic | 5 | - | - | - | Bright |
| S65 | SpeaKWeb | Synthetic | 5 | - | - | - | Continuous |
| S66 | SpeaKWeb | Environmental | 3.66 | - | - | - | Discontinuous |
| S67 | SpeaKWeb | Synthetic | 5 | - | - | - | Fluctuating |
| S68 | SpeaKWeb | Environmental | 5.44 | - | - | - | Resonant |
| S69 | SpeaKWeb | Instrumental | 3.30 | - | - | - | Resonant |
| S70 | SpeaKWeb | Instrumental | 5 | - | - | - | Rough |
| S71 | VSL | Instrumental | 3 | Woodwind | English Horn | 4 | - |
| S72 | VSL | Instrumental | 3 | Strings | Violin | 5 | - |
| S73 | VSL | Instrumental | 3 | Strings | Violin | 6 | - |
| S74 | SpeaKWeb | Environmental | 6.94 | - | - | - | Artificial |
| S75 | SpeaKWeb | Synthetic | 5.5 | - | - | - | Ascending |
| S76 | SpeaKWeb | Synthetic | 5 | - | - | - | Warm |
| S77 | SpeaKWeb | Synthetic | 5 | - | - | - | Metallic |
| S78 | SpeaKWeb | Synthetic | 5 | - | - | - | Non-rich |
| S79 | SpeaKWeb | Instrumental | 5 | - | - | - | Rough |
| S80 | SpeaKWeb | Synthetic | 5 | - | - | - | Rough |
