# Supplementary material for: The sound of smell: crossmodal correspondences between sound and odor
Source: Front Psychol. 2026 Sep 18;17:1890636. doi: 10.3389/fpsyg.2026.1890636 (PMC13631861; doi:10.3389/fpsyg.2026.1890636)
Supplement: Supplementary file 3 [file Table_2.docx]

Supplementary Table 2. General characteristics of the odor stimuli used in Experiment 2.
Each odor stimulus is described by its origin and olfactory family. No statistically robust associations between olfactory family and affect were observed after accounting for the limited sample size (n = 24) and the exploratory nature of the analysis; therefore, these findings should not be considered generalizable.

| Odor/Ingredient | Synthetic/Natural | Olfactory family |
| --- | --- | --- |
| I1 | Synthetic | Floral |
| I2 | Synthetic | Green & Moss |
| I3 | Synthetic | Citrus |
| I4 | Synthetic | Musk |
| I5 | Natural | Balsam |
| I6 | Synthetic | Woody |
| I7 | Synthetic | Fruity |
| I8 | Synthetic | Musk |
| I9 | Natural | Herbal |
| I10 | Natural | Spice |
| I11 | Synthetic | Leather |
| I12 | Synthetic | Woody |
| I13 | Synthetic | Musk |
| I14 | Synthetic | Musk |
| I15 | Synthetic | Sweet |
| I16 | Natural | Floral |
| I17 | Synthetic | Sweet |
| I18 | Synthetic | Musk |
| I19 | Synthetic | Citrus |
| I20 | Natural | Herbal |
| I21 | Synthetic | Fruity |
| I22 | Synthetic | Aldehydic |
| I23 | Synthetic | Green |
| I24 | Synthetic | Floral |
