# Supplementary material for: The sound of smell: crossmodal correspondences between sound and odor
Source: Front Psychol. 2026 Sep 18;17:1890636. doi: 10.3389/fpsyg.2026.1890636 (PMC13631861; doi:10.3389/fpsyg.2026.1890636)
Supplement: Supplementary file 4 [file Table_3.docx]

**Supplementary Table 3.** Contingency table for Experiment 1 reporting the frequency with which each combination of stimulus × affect occurs. Each cell indicates how many participants associated a given sound with one of the eight affects. Cell values range from 0 to 59 (61 participants minus 2 excluded outliers), where 0 means that no participant selected that affect for that sound and 59 means that all participants did so.

| Sound | Apathy | Depression | Happiness | Irritation | Relaxation | Sensuality | Stimulation | Stress |
| --- | --- | --- | --- | --- | --- | --- | --- | --- |
| S1 | 18 | 14 | 0 | 5 | 4 | 0 | 0 | 0 |
| S2 | 0 | 8 | 2 | 5 | 1 | 0 | 0 | 4 |
| S3 | 3 | 3 | 2 | 6 | 4 | 2 | 6 | 6 |
| S4 | 10 | 11 | 4 | 5 | 16 | 2 | 0 | 8 |
| S5 | 3 | 0 | 15 | 3 | 1 | 6 | 15 | 5 |
| S6 | 5 | 29 | 4 | 3 | 2 | 5 | 1 | 4 |
| S7 | 26 | 9 | 2 | 5 | 30 | 4 | 4 | 8 |
| S8 | 11 | 19 | 4 | 4 | 26 | 13 | 4 | 5 |
| S9 | 11 | 1 | 1 | 4 | 1 | 0 | 0 | 1 |
| S10 | 10 | 16 | 2 | 8 | 7 | 6 | 7 | 16 |
| S11 | 16 | 13 | 0 | 8 | 2 | 0 | 1 | 3 |
| S12 | 14 | 15 | 0 | 2 | 3 | 1 | 0 | 1 |
| S13 | 18 | 9 | 0 | 12 | 0 | 0 | 0 | 4 |
| S14 | 15 | 20 | 0 | 13 | 4 | 2 | 2 | 6 |
| S15 | 9 | 9 | 0 | 5 | 8 | 2 | 1 | 9 |
| S16 | 7 | 11 | 0 | 4 | 3 | 0 | 1 | 2 |
| S17 | 11 | 16 | 0 | 14 | 1 | 0 | 2 | 9 |
| S18 | 12 | 30 | 4 | 1 | 17 | 11 | 0 | 4 |
| S19 | 12 | 42 | 0 | 0 | 10 | 11 | 1 | 1 |
| S20 | 6 | 10 | 0 | 3 | 1 | 1 | 1 | 2 |
| S21 | 1 | 5 | 13 | 0 | 16 | 11 | 7 | 0 |
| S22 | 2 | 0 | 20 | 1 | 9 | 3 | 19 | 1 |
| S23 | 1 | 0 | 8 | 0 | 4 | 1 | 11 | 0 |
| S24 | 0 | 0 | 21 | 0 | 10 | 4 | 12 | 2 |
| S25 | 1 | 0 | 34 | 1 | 20 | 36 | 8 | 1 |
| S26 | 5 | 6 | 22 | 2 | 5 | 12 | 8 | 3 |
| S27 | 10 | 6 | 5 | 5 | 0 | 5 | 6 | 4 |
| S28 | 1 | 6 | 11 | 8 | 9 | 7 | 5 | 11 |
| S29 | 1 | 1 | 21 | 4 | 0 | 13 | 43 | 13 |
| S30 | 1 | 0 | 7 | 4 | 0 | 5 | 8 | 0 |
| S31 | 2 | 2 | 7 | 18 | 0 | 1 | 5 | 23 |
| S32 | 1 | 3 | 16 | 0 | 10 | 8 | 16 | 2 |
| S33 | 0 | 3 | 5 | 25 | 0 | 2 | 9 | 21 |
| S34 | 2 | 2 | 22 | 8 | 0 | 7 | 17 | 13 |
| S35 | 13 | 11 | 0 | 20 | 0 | 0 | 1 | 8 |
| S36 | 2 | 4 | 6 | 28 | 0 | 0 | 3 | 18 |
| S37 | 1 | 0 | 4 | 15 | 0 | 3 | 18 | 29 |
| S38 | 5 | 3 | 7 | 42 | 0 | 0 | 7 | 28 |
| S39 | 2 | 8 | 1 | 32 | 0 | 0 | 8 | 41 |
| S40 | 4 | 0 | 8 | 25 | 0 | 2 | 18 | 23 |
| S41 | 2 | 3 | 2 | 0 | 11 | 1 | 6 | 2 |
| S42 | 0 | 0 | 15 | 3 | 11 | 3 | 18 | 3 |
| S43 | 12 | 6 | 1 | 4 | 13 | 7 | 1 | 3 |
| S44 | 27 | 24 | 0 | 4 | 21 | 6 | 0 | 3 |
| S45 | 0 | 0 | 30 | 1 | 18 | 29 | 14 | 0 |
| S46 | 19 | 17 | 1 | 4 | 37 | 12 | 3 | 4 |
| S47 | 10 | 9 | 2 | 0 | 21 | 3 | 0 | 1 |
| S48 | 9 | 4 | 3 | 4 | 11 | 1 | 0 | 0 |
| S49 | 0 | 4 | 16 | 6 | 8 | 15 | 3 | 3 |
| S50 | 16 | 4 | 4 | 2 | 17 | 10 | 2 | 1 |
| S51 | 14 | 14 | 3 | 2 | 5 | 0 | 0 | 1 |
| S52 | 20 | 16 | 0 | 7 | 3 | 1 | 0 | 4 |
| S53 | 9 | 12 | 8 | 1 | 32 | 18 | 1 | 1 |
| S54 | 10 | 18 | 0 | 5 | 1 | 0 | 0 | 4 |
| S55 | 4 | 1 | 7 | 0 | 23 | 5 | 3 | 1 |
| S56 | 10 | 15 | 5 | 2 | 19 | 8 | 0 | 10 |
| S57 | 26 | 13 | 2 | 4 | 16 | 8 | 2 | 1 |
| S58 | 33 | 9 | 2 | 8 | 18 | 6 | 2 | 4 |
| S59 | 35 | 19 | 0 | 6 | 13 | 4 | 1 | 8 |
| S60 | 8 | 5 | 9 | 0 | 22 | 6 | 2 | 0 |
| S61 | 3 | 10 | 11 | 1 | 0 | 5 | 5 | 4 |
| S62 | 1 | 2 | 9 | 2 | 2 | 3 | 11 | 6 |
| S63 | 1 | 1 | 14 | 14 | 1 | 7 | 15 | 6 |
| S64 | 1 | 1 | 10 | 16 | 2 | 7 | 31 | 26 |
| S65 | 2 | 0 | 22 | 3 | 0 | 6 | 42 | 9 |
| S66 | 3 | 1 | 2 | 20 | 2 | 3 | 5 | 23 |
| S67 | 0 | 0 | 49 | 2 | 2 | 9 | 46 | 5 |
| S68 | 1 | 0 | 13 | 3 | 8 | 1 | 8 | 0 |
| S69 | 1 | 0 | 15 | 4 | 0 | 2 | 12 | 4 |
| S70 | 1 | 3 | 2 | 32 | 0 | 0 | 16 | 38 |
| S71 | 4 | 9 | 1 | 6 | 2 | 0 | 1 | 5 |
| S72 | 3 | 15 | 1 | 14 | 0 | 1 | 0 | 13 |
| S73 | 2 | 10 | 1 | 24 | 1 | 1 | 1 | 17 |
| S74 | 0 | 3 | 1 | 28 | 2 | 1 | 7 | 26 |
| S75 | 2 | 0 | 11 | 9 | 0 | 5 | 27 | 8 |
| S76 | 6 | 4 | 2 | 18 | 5 | 2 | 10 | 25 |
| S77 | 1 | 2 | 5 | 10 | 1 | 6 | 17 | 24 |
| S78 | 1 | 2 | 6 | 9 | 0 | 7 | 25 | 24 |
| S79 | 8 | 5 | 0 | 30 | 0 | 1 | 0 | 27 |
| S80 | 1 | 0 | 2 | 24 | 0 | 1 | 15 | 28 |
