# Supplementary material for: The sound of smell: crossmodal correspondences between sound and odor
Source: Front Psychol. 2026 Sep 18;17:1890636. doi: 10.3389/fpsyg.2026.1890636 (PMC13631861; doi:10.3389/fpsyg.2026.1890636)
Supplement: Supplementary file 5 [file Table_4.docx]

**Supplementary Table 4.** Contingency table for Experiment 2 reporting the frequency with which each combination of stimulus × affect occurs. Each cell indicates how many participants associated a given odor with one of the eight affects. Cell values range from 0 to 60, where 0 indicates that no participant selected that affect for that odor and 60 indicates that all participants did so.

| Odor | Apathy | Depression | Happiness | Irritation | Relaxation | Sensuality | Stimulation | Stress |
| --- | --- | --- | --- | --- | --- | --- | --- | --- |
| I1 | 9 | 16 | 2 | 27 | 3 | 2 | 2 | 25 |
| I2 | 19 | 15 | 6 | 8 | 6 | 5 | 4 | 9 |
| I3 | 10 | 10 | 1 | 20 | 1 | 0 | 7 | 26 |
| I4 | 13 | 15 | 3 | 25 | 4 | 2 | 3 | 27 |
| I5 | 14 | 14 | 4 | 6 | 4 | 4 | 0 | 9 |
| I6 | 23 | 11 | 4 | 1 | 16 | 10 | 1 | 4 |
| I7 | 4 | 2 | 22 | 6 | 6 | 10 | 33 | 5 |
| I8 | 8 | 7 | 29 | 2 | 25 | 24 | 5 | 4 |
| I9 | 1 | 6 | 18 | 2 | 29 | 10 | 17 | 4 |
| I10 | 7 | 5 | 5 | 17 | 4 | 4 | 23 | 8 |
| I11 | 13 | 14 | 1 | 23 | 3 | 0 | 5 | 24 |
| I12 | 19 | 14 | 4 | 14 | 5 | 8 | 7 | 12 |
| I13 | 15 | 15 | 9 | 0 | 15 | 23 | 4 | 2 |
| I14 | 12 | 13 | 18 | 0 | 22 | 26 | 9 | 3 |
| I15 | 8 | 5 | 23 | 7 | 13 | 13 | 6 | 6 |
| I16 | 6 | 10 | 24 | 7 | 21 | 21 | 12 | 4 |
| I17 | 10 | 7 | 13 | 3 | 21 | 15 | 2 | 3 |
| I18 | 14 | 14 | 13 | 1 | 21 | 20 | 10 | 6 |
| I19 | 4 | 1 | 34 | 2 | 14 | 14 | 41 | 0 |
| I20 | 6 | 10 | 10 | 12 | 11 | 4 | 27 | 14 |
| I21 | 3 | 6 | 7 | 17 | 1 | 6 | 17 | 17 |
| I22 | 3 | 9 | 0 | 26 | 1 | 1 | 8 | 22 |
| I23 | 8 | 7 | 3 | 13 | 3 | 2 | 5 | 14 |
| I24 | 7 | 7 | 2 | 20 | 0 | 1 | 5 | 21 |
