# Supplementary material for: The sound of smell: crossmodal correspondences between sound and odor
Source: Front Psychol. 2026 Sep 18;17:1890636. doi: 10.3389/fpsyg.2026.1890636 (PMC13631861; doi:10.3389/fpsyg.2026.1890636)
Supplement: Supplementary file 8 [file Table_7.docx]

**Supplementary Table 6.** Details on the odors used in Experiment 3 (timbre × odor), including their type, association scores with each affect from Experiment 2, and the affect they represent in Experiment 3.

| Ingredient/  Odor | Type | Apathy | Depression | Happiness | Irritation | Relaxation | Sensuality | Stimulation | Stress |
| --- | --- | --- | --- | --- | --- | --- | --- | --- | --- |
| Apathy Odor Group | | | | | | | | | |
| I6 | Synthetic | 23 | 11 | 4 | 1 | 16 | 10 | 1 | 4 |
| I12 | Synthetic | 19 | 14 | 4 | 14 | 5 | 8 | 7 | 12 |
| Happiness Odor Group | | | | | | | | | |
| I15 | Synthetic | 8 | 5 | 23 | 7 | 13 | 13 | 6 | 6 |
| I16 | Natural | 6 | 10 | 24 | 7 | 21 | 21 | 12 | 4 |
| Relaxation Odor Group | | | | | | | | | |
| I14 | Synthetic | 12 | 13 | 18 | 0 | 22 | 26 | 9 | 3 |
| I17 | Synthetic | 10 | 7 | 13 | 3 | 21 | 15 | 2 | 3 |
| Stimulation Odor Group | | | | | | | | | |
| I7 | Synthetic | 4 | 2 | 22 | 6 | 6 | 10 | 33 | 5 |
| I19 | Synthetic | 4 | 1 | 34 | 2 | 14 | 14 | 41 | 0 |
| Stress /Irritation Odor Group | | | | | | | | | |
| I11 | Synthetic | 13 | 14 | 1 | 23 | 3 | 0 | 5 | 24 |
| I22 | Synthetic | 3 | 9 | 0 | 26 | 1 | 1 | 8 | 22 |
